# VAV-1 acts in a single interneuron to inhibit motor circuit activity in *Caenorhabditis elegans*

**DOI:** 10.1038/ncomms6579

**Published:** 2014-11-21

**Authors:** Amanda L. Fry, Jocelyn T. Laboy, Kenneth R. Norman

**Affiliations:** 1 Center for Cell Biology and Cancer Research, Albany Medical College, Albany, New York 12208, USA

## Abstract

The complex molecular and cellular mechanisms underlying neuronal control of animal movement are not well understood. Locomotion of *Caenorhabditis elegans* is mediated by a neuronal circuit that produces coordinated sinusoidal movement. Here we utilize this simple, yet elegant, behaviour to show that VAV-1, a conserved guanine nucleotide exchange factor for Rho-family GTPases, negatively regulates motor circuit activity and the rate of locomotion. While *vav-1* is expressed in a small subset of neurons, we find that VAV-1 function is required in a single interneuron, ALA, to regulate motor neuron circuit activity. Furthermore, we show by genetic and optogenetic manipulation of ALA that VAV-1 is required for the excitation and activation of this neuron. We find that ALA signalling inhibits command interneuron activity by abrogating excitatory signalling in the command interneurons, which is responsible for promoting motor neuron circuit activity. Together, our data describe a novel neuromodulatory role for VAV-1-dependent signalling in the regulation of motor circuit activity and locomotion.

## Abstract (web summary)

VAV proteins are guanine nucleotide exchange factors that have various neuromodulatory roles in the nervous system. Here, Fry *et al.* show that in *Caenorhabditis elegans*, VAV-1 negatively regulates locomotor speed and only needs to be expressed in a specific interneuron to do so.

## Graphical abstract

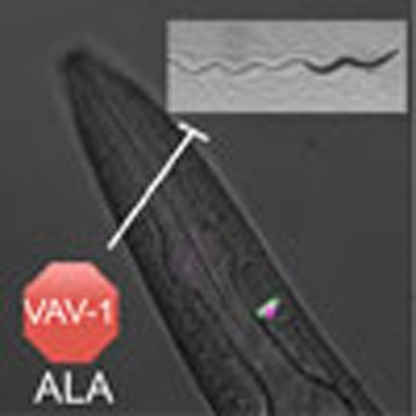

Vav proteins are a family of evolutionarily conserved molecules that contain a series of protein–protein interaction domains, including SH2 and SH3 domains, and a Dbl homology domain that acts as a Rho GTPase family guanine nucleotide exchange factor (GEF). The first identified Vav protein, Vav1, is highly expressed in the immune system, whereas Vav2 and Vav3 are ubiquitous and are highly expressed in the nervous system^1^,^2^,^3^,^4^. While the function of Vav in the immune system has been well studied, the understanding of Vav proteins in the nervous system is limited. Nevertheless, Vav proteins have been implicated in axonal growth and guidance, cerebellar development and plasticity^3^,^4^,^5^,^6^. Furthermore, Vav2 and Vav3 single-knockout mice exhibit evidence of sympathetic neuron hyperactivity, including elevated release of noradrenaline, adrenaline and dopamine^7^,^8^, suggesting Vav proteins have a neuromodulatory role in the nervous system.

To explore the molecular mechanisms underlying the regulation of behaviour, we are studying locomotion in the model system *Caenorhabditis elegans*. *C. elegans* locomotion is governed by a small network of command interneurons that are postsynaptic to glutamatergic sensory neurons and presynaptic to the motor neurons that drive locomotion (Fig. 1a)^9^,^10^,^11^,^12^. Excitatory signalling from the sensory neurons to the command interneurons promotes locomotion^13^,^14^. This simple locomotory control circuit and the fact that the *C. elegans* genome encodes only one Vav family member, *vav-1* (ref. 15) make *C. elegans* a valuable experimental system to help dissect the role of Vav proteins in the nervous system. Previously, *vav-1* was shown to regulate several rhythmic behaviours^15^,^16^,^17^. Here, we show that loss of *vav-1* function disrupts normal motor circuit activity. While *vav-1* mutants exhibit normal sinusoidal locomotion and nervous system development, they display increased locomotory velocity and elevated motor circuit activity. Moreover, by restoring VAV-1 expression in a single interneuron (ALA), the elevated motor circuit activity of *vav-1* mutants can be returned to a wild-type (WT) level. Finally, we have found evidence that VAV-1-dependent signalling in ALA opposes the activity of the command interneurons that directly promote motor circuit activity and animal locomotion (Fig. 1a). Together, our results suggest that VAV-1 has a crucial role in regulating a neural circuit required for controlling motor neuron activity, which controls the rhythm of sinusoidal locomotion.

## Results

### *vav-1* mutants have an elevated rate of locomotion

**Figure 1:**
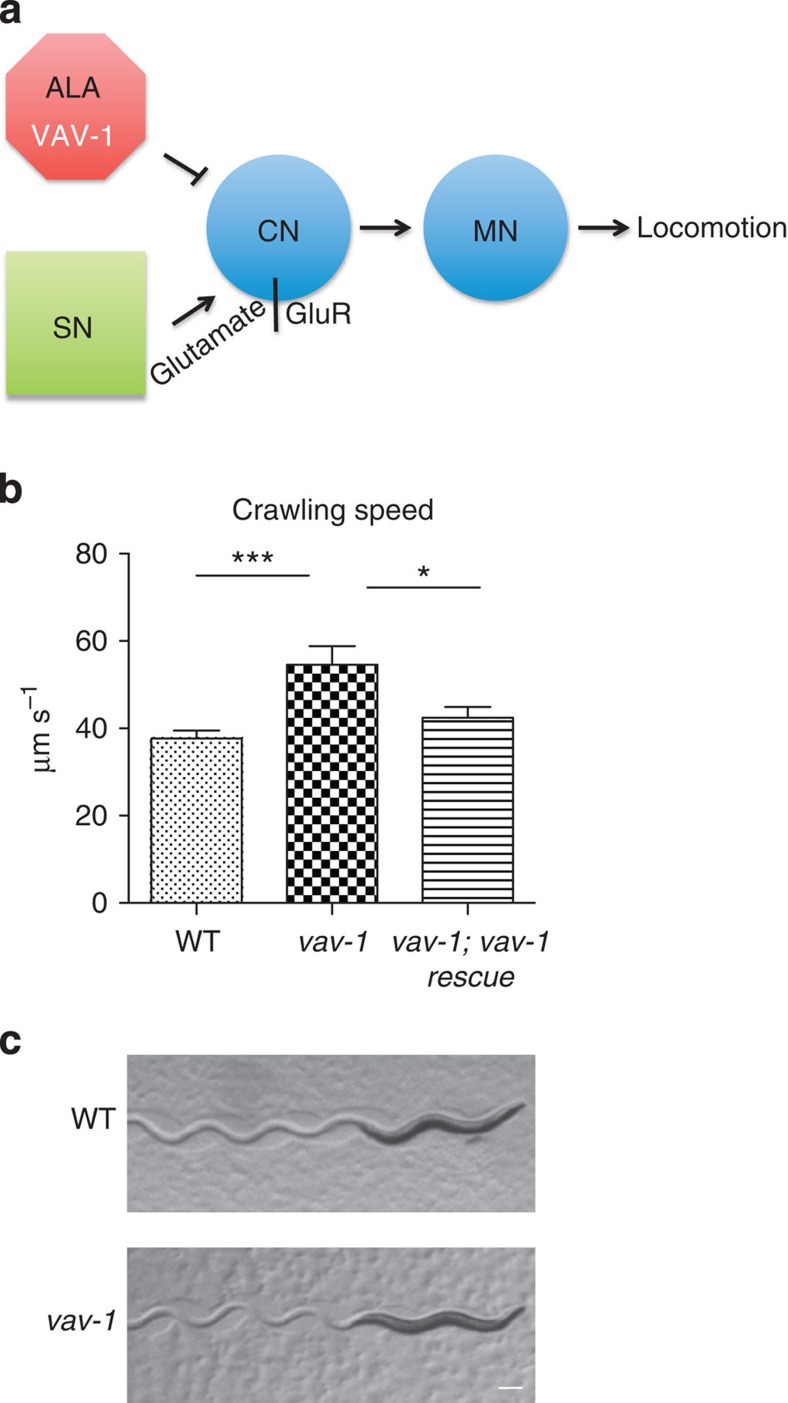
*vav-1* mutant worms are hyperactive. (**a**) Schematic of the locomotory control circuit (motor circuit) utilized by *C. elegans*. Glutamatergic sensory neurons (SNs) stimulate the activity of a set of command interneurons (CNs), which include AVA, AVD, AVE and PVC (GluR, glutamate receptor). Activation of the command interneurons promotes locomotion by direct regulation of the motor neurons (MNs). VAV-1 acts in ALA to inhibit motor circuit activity by blocking command interneuron activity. (**b**) *vav-1* mutants crawl faster than wild-type (WT) animals on an agar surface seeded with bacteria, which is rescued by VAV-1 expression under control of the full *vav-1* promoter. *n*=5–10 animals in 7 (*vav-1; vav-1 rescue*), 9 (*vav-1*) or 11 (WT) replicate assays. Data are represented as mean ±s.e.m. (***P<0.001, *P<0.05 using one-way analysis of variance). (**c**) WT and *vav-1* mutants exhibit characteristic coordinated forward sinusoidal locomotion. Anterior is to the right. Scale bar, 100 μm.

In *C. elegans*, loss-of-function mutations in *vav-1* result in severely defective pharyngeal pumping, which leads to larval lethality, since animals cannot feed. Selective expression of *vav-1* in pharyngeal tissue completely restores pharyngeal activity and animals develop into sexually mature adults^15^. However, in rescued *vav-1* mutant animals, referred to as *vav-1* mutants from this point forward, defects in other tissues are observed, including abnormal ovulation, fertilization and defecation cycle timing^15^. While documenting these adult phenotypes, we observed that *vav-1* mutants are more active than WT worms. To quantify the locomotory behaviour of *vav-1* mutants, we measured the crawling speed of animals moving on an agar surface seeded with bacteria. Consistent with our observations, *vav-1* mutants show an increase in crawling velocity compared with WT animals (Fig. 1b). To determine whether this elevated locomotion was the result of disrupted VAV-1 function, we introduced a WT *vav-1* genomic construct into *vav-1* mutants and found that this rescues the elevated locomotion phenotype (Fig. 1b). Importantly, although *vav-1* mutants travel faster than WT worms, they maintain a normal sinusoidal mode of locomotion and normal posture (Fig. 1c), indicating coordinated motor output.

### *vav-1* mutants have elevated motor circuit activity

**Figure 2:**
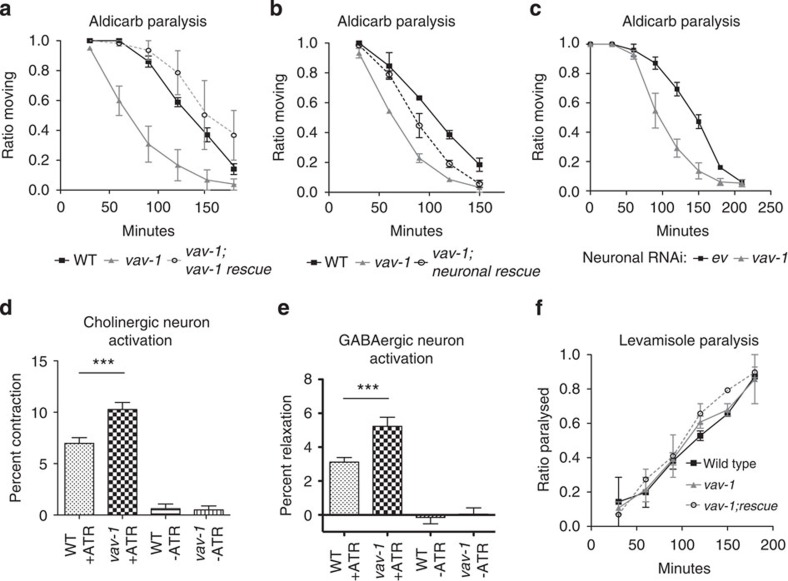
*vav-1* mutants exhibit elevated motor neuron excitability. (**a**,**b**) Animals were paralysed with 1 mM aldicarb. (**a**) *vav-1* mutants are hypersensitive to aldicarb, and *vav-1* expression driven by the full *vav-1* promoter (*vav-1; vav-1 rescue*) rescues this hypersensitivity. (**b**) Rescue of *vav-1* mutant aldicarb hypersensitivity by a pan-neuronal promoter, *aex-3* (*vav-1; neuronal rescue*) is shown. (**c**) Neuronal-specific RNAi of *vav-1* causes hypersensitivity in a 0.5 mM aldicarb paralysis assay, similar to the effect of *vav-1* mutation. Control: empty vector (*ev*). For **a**–**c**, *n*=15–20 animals in ≥5 replicate assays. (**d**,**e**) Cholinergic neurons or GABAergic neurons were specifically activated using channelrhodopsin (ChR2). (**d**) *vav-1* mutant worms contract significantly more than WT upon cholinergic stimulation (percent change of pre-stimulus worm length). *n*=48 (+ATR) or 27 (−ATR controls) animals per genotype. (**e**) *vav-1* mutant worms relax significantly more than WT upon GABAergic stimulation (percent change of pre-stimulus worm length). *n*=37 (+ATR) or 27 (−ATR) animals per genotype. Data are displayed as mean ±s.e.m. (***P<0.001 using two-tailed *T*-tests). (**f**) Animals were paralysed with 100 μM levamisole. *vav-1* mutants show a normal muscle response to levamisole. Aldicarb and levamisole paralysis curves display the mean proportions of moving or paralysed worms, ±s.e.m. (statistical significance determined by two-way analysis of variance and Bonferroni post-tests; see the Methods section for detailed analysis).

Since Vav proteins are expressed in the nervous system, we hypothesized that VAV-1 acts in the nervous system to regulate motor activity. To investigate the role VAV-1 may have in the nervous system, we employed a pharmacological assay using the small-molecule aldicarb. Aldicarb is an acetylcholine (ACh) esterase inhibitor, which prevents the breakdown of the excitatory neurotransmitter ACh at synapses of neuromuscular junctions (NMJs)^18^. In *C. elegans*, exposure to aldicarb leads to progressive paralysis over time^18^. Moreover, mutations that increase synaptic transmission cause hypersensitivity to aldicarb, and those that decrease synaptic transmission cause resistance to the drug^18^. Strikingly, we found that *vav-1* mutants are hypersensitive to aldicarb and become paralysed more rapidly than WT animals, indicating excessive synaptic transmission (Fig. 2a). Similar to the hyperactive locomotion detected in *vav-1* mutants, the increased sensitivity to aldicarb can be rescued by expression of a WT *vav-1* genomic fragment in *vav-1* mutant animals (Fig. 2a). Importantly, aldicarb hypersensitivity is consistent with the increased rate of locomotion observed in *vav-1* mutants, suggesting that VAV-1 negatively regulates motor circuit activity. Thus, to test whether *vav-1* activity is required in the nervous system, we selectively expressed the *vav-1* complementary DNA in the nervous system of *vav-1* mutants using a pan-neuronal promoter from the *aex-3* gene^19^. Accordingly, we found that neuronal expression of *vav-1* can rescue the aldicarb hypersensitivity caused by loss of *vav-1* (Fig. 2b). Similarly, we found that knocking down VAV-1 expression by neuronal-specific RNA interference (RNAi) causes aldicarb hypersensitivity similar to that of *vav-1* mutant worms (Fig. 2c). These data suggest that *vav-1* activity is required in the nervous system to inhibit motor circuit activity.

To further explore the neuronal function of VAV-1, we utilized transgenic animals that express channelrhodopsin-2 (ChR2) specifically in cholinergic (excitatory) and GABAergic (inhibitory) motor neurons. ChR2s are light-gated ion channels that nonspecifically allow the flow of cations into a cell, leading to electrical excitation^20^. Thus, activation of ChR2 in cholinergic neurons results in a visible and measurable body wall muscle contraction as quantified by the percent change in overall length of the animal (Fig. 2d)^20^,^21^. In contrast, activation of ChR2 in the GABAergic neurons results in a visible and measurable body wall muscle relaxation (Fig. 2e)^21^. Activation of cholinergic ChR2 in the *vav-1* mutant background results in enhanced muscle contraction compared with WT animals (Fig. 2d). In addition, we found that activation of GABAergic ChR2 results in enhanced relaxation in the *vav-1* mutant background compared with the WT background (Fig. 2e). These findings suggest that *vav-1* mutants have hyperexcitable motor neurons, and further indicate that VAV-1 may play an inhibitory role in regulating motor circuit activity.

However, it is possible that the *vav-1* mutant aldicarb hypersensitivity and excessive response to ChR2 activation are caused by muscle cell defects. For instance, heightened muscle cell response to neurotransmitters could explain these phenotypes. Therefore, we tested the sensitivity of *vav-1* mutants to levamisole, an ACh receptor agonist. Similar to aldicarb, levamisole causes paralysis over time. Levamisole activates postsynaptic muscle cells, so paralysis occurs independently of presynaptic (neuronal) defects^18^. Analysis of levamisole-treated animals revealed that *vav-1* mutants responded like WT animals (Fig. 2f), indicating normal activity of muscle in *vav-1* mutants. Taken together, our data indicate that VAV-1 acts in the nervous system to regulate motor circuit activity.

Since internal metabolic signals resulting from being well-fed or starved can influence the rate of locomotion of *C. elegans*^22^, and mutations in genes affecting feeding can lead to aldicarb hypersensitivity^23^, it is possible that *vav-1* mutants do not feed as well as WT animals, resulting in enhanced locomotion and aldicarb sensitivity. However, this is unlikely because mutant *vav-1* animals develop at a similar rate as WT animals; we regularly obtain synchronized populations of adult WT and *vav-1* animals for use in assays described here. Furthermore, we investigated fat accumulation in adult *vav-1* mutants by Oil-Red-O staining^24^ and found that *vav-1* mutants were indistinguishable from WT animals (Supplementary Fig. 1). These data indicate that *vav-1* mutants develop at a similar rate and have similar metabolic activity as WT animals.

### *vav-1* is expressed in different classes of neurons

**Figure 3:**
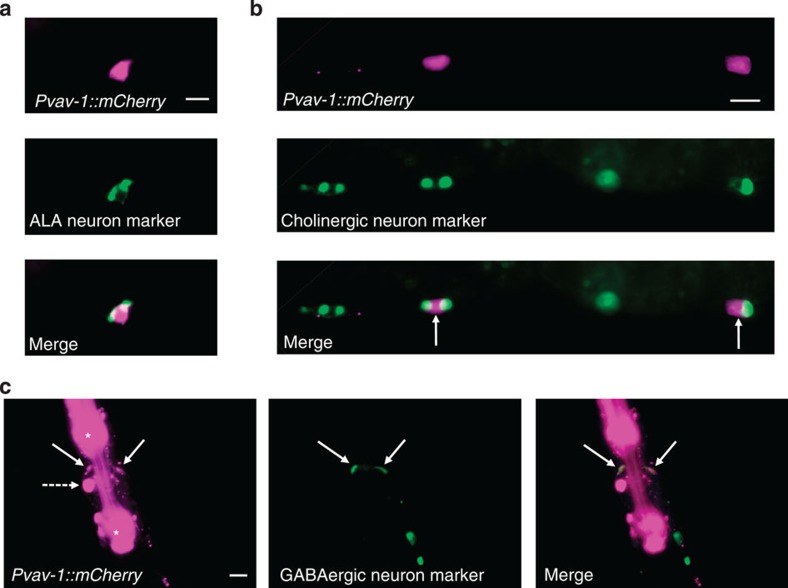
VAV-1 is expressed in multiple neuronal cell types. (**a**–**c**) Expression of mCherry, tagged with a 2 × -nuclear localization signal, under control of the *vav-1* promoter, is shown. (**a**) *vav-1* reporter expression co-localizes with a GFP marker for the ALA interneuron. Anterior is to the left. (**b**) *vav-1* reporter expression is found in cholinergic motor neurons of the ventral nerve cord. Shown are two posterior cholinergic motor neurons (VA11 and VA12) and their co-localization with cytoplasmic cholinergic GFP reporter. Arrows mark these two cell bodies. Anterior is to the left. (**c**) Only two GABAergic neurons (RME dorsal and ventral, head motor neurons) express the *vav-1* reporter. Asterisks mark *vav-1* expression in the pharynx, a dashed arrow marks the ALA neuron and solid arrows mark the two RME neurons that express the *vav-1* reporter and co-localize with a GABA neuron-specific GFP marker. Anterior is up. Scale bars, 5 μm.

To determine which neurons may require VAV-1 function for normal locomotion and motor circuit activity, we used a standard reporter gene fusion approach^25^ to express fluorescent proteins (green fluorescent protein (GFP) and mCherry) under control of the *vav-1* promoter^15^. For this analysis, we used the same genomic sequence for the *vav-1* promoter that rescued the elevated crawling speed and aldicarb hypersensitivity of *vav-1* mutants when driving expression of WT *vav-1* (Figs 1b and 2a). The fluorescent proteins were targeted to the nucleus by a nuclear localization signal to aid in cell identification. We observed fluorescence in expected tissues, such as the pharynx and intestine^15^, as well as in a small set of neurons. In five independent reporter lines generated, fluorescence was regularly observed in the ALA interneuron. To confirm the identity of this cell, we crossed a *vav-1* mCherry reporter line into animals carrying an ALA neuron marker, IDA-1::GFP, and observed co-localization (Fig. 3a). Interestingly, the ALA interneuron has been implicated in regulating sleep-like behaviour^26^. In addition, we observed variable reporter expression in cholinergic motor neurons in the ventral nerve cord (Fig. 3b), which are required for locomotion, but did not observe expression in GABA motor neurons of the ventral nerve cord. Rather, the *vav-1* reporter was expressed in two GABA motor neurons in the head, known as RME dorsal and RME ventral, which regulate head oscillations (Fig. 3c). Several cell types were found to be negative for *vav-1* reporter expression. We found that our *vav-1* reporters did not show co-expression with markers of dopaminergic neurons (*Pdat-1::GFP*)^27^, command interneurons of the locomotory circuit, which directly activate motor neurons (*Pglr-1::RFP*)^28^ or with a marker of a small set of sensory neurons (AWC and ASE neurons, marked by *Pceh-36::RFP*)^29^ (Supplementary Fig. 2). In sum, fluorescent *vav-1* reporters are expressed in a small subset of neurons that comprise multiple cell types, including an interneuron, cholinergic motor neurons and a small subset of GABAergic motor neurons.

### VAV-1 is not required for neuronal development

**Figure 4:**
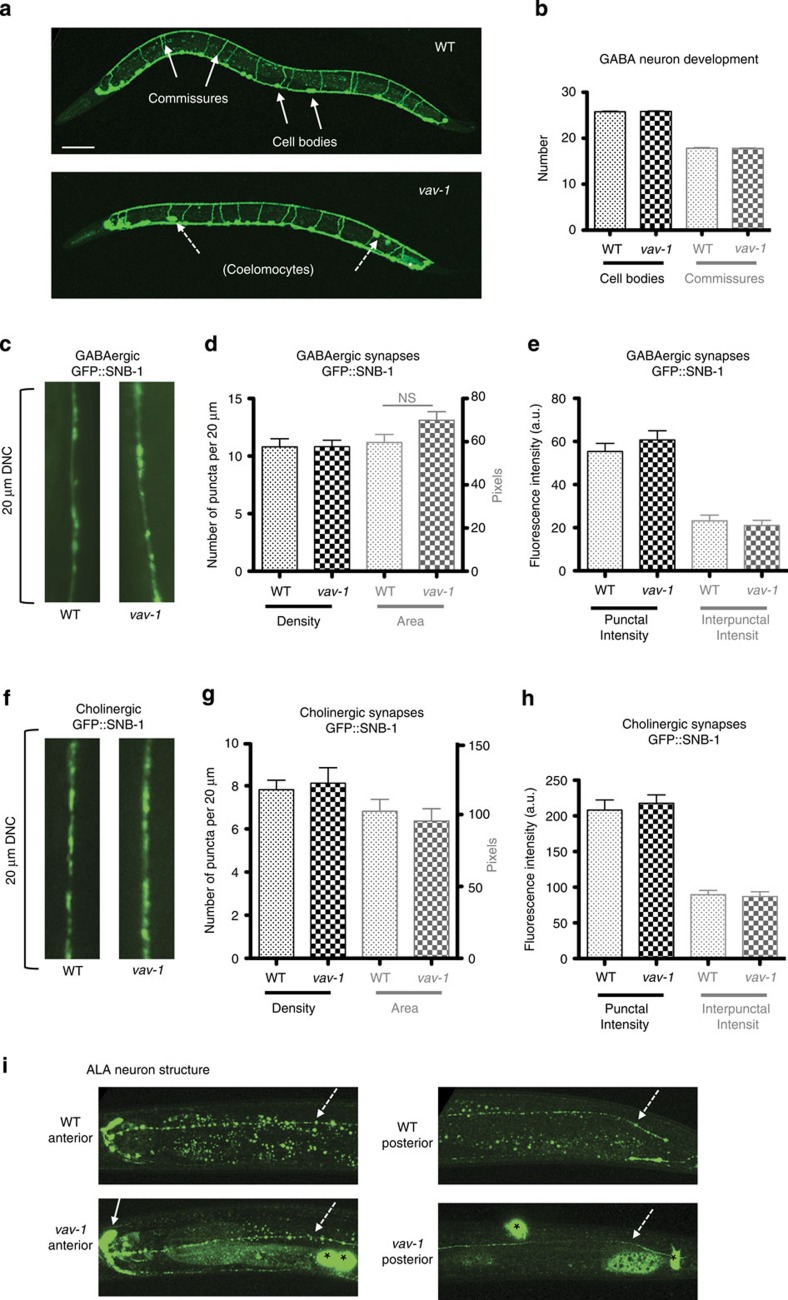
Loss of VAV-1 does not lead to defects in neuronal development or synapse formation. (**a**) Representative confocal micrographs of WT (top panel) and *vav-1* mutant (bottom panel) animals expressing GFP in all GABAergic neurons (*oxIs12 [Punc-47::GFP]*). In WT (top panel), arrows and lines point to GABA neuron representative cell bodies and circumferential axons (commissures), respectively. In *vav-1* mutants (bottom panel), these features are also present. Dashed arrows indicate fluorescent coelomocytes, a marker of the *vav-1* mutant strain that should be ignored. Asterisk marks non-specific posterior intestinal GFP. Anterior is to the left, and scale bars, 100 μm. (**b**) Quantification of the number of GABA neuron cell bodies and commissures indicates that *vav-1* mutants show grossly normal GABA neuron development. *n*=13 (WT) and 19 (*vav-1*). (**c**) Representative images of GABAergic neuron synapses and (**f**) cholinergic synapses in the dorsal nerve cord of WT (left) and *vav-1* mutant (right); fluorescent puncta are synaptobrevin tagged with GFP at the neuromuscular junction (NMJ). GABAergic animals are *juIs1 [Punc-25::GFP::snb-1]*, and cholinergic are *nuIs152 [Punc-129::GFP::snb-1]*. (**d**,**g**) Quantification of density of fluorescent puncta (left *y* axis) and the mean area of puncta (right *y* axis). For **d**, *n*=10 (WT) and 11 (*vav-1*). For **g**, *n*=11 (WT) and 16 (*vav-1*). (**e**,**h**) Quantification of fluorescence intensity of puncta (left *y* axis), and of nerve cord between puncta (right *y* axis). For **e**, *n*=10 (WT) and 11 (*vav-1*). For **h**, *n*=28 (WT) and 36 (*vav-1*). (**i**) The structure of the ALA neuron in WT and *vav-1* mutants is shown using a fluorescent ALA reporter analysed by confocal microscopy (*Pida-1::ida-1::GFP*). In the left panels, solid arrows point to the ALA neuron cell body and a dashed arrow points to one ALA axonal projection. The right panels show the same axons projecting to the tails of these animals. Asterisks mark GFP associated with coelomocytes and rectal epithelial cells, and can be ignored. *n*=3(WT) and 11 (*vav-1*). Quantified data are displayed as mean ±s.e.m. and were analysed by two-tailed Student’s *T*-tests (no significant differences were found). NS, not significant.

Previous studies have indicated that Vav proteins in *Drosophila* and in mice are important for neuronal development^3^,^4^,^5^. Specifically, these studies have shown that Vav proteins are required for axonal guidance and cell migration events in the developing nervous system. Since defects in production or release of the inhibitory neurotransmitter GABA can cause aldicarb hypersensitivity due to increased excitability at the NMJ^30^,^31^, we reasoned that improper development (for example, differentiation, axon guidance or cell migration) of GABAergic neurons or impaired GABA release could explain the aldicarb hypersensitivity phenotype of *vav-1* mutants (Fig. 2a). Although we did not observe *vav-1* reporter expression in ventral nerve cord GABAergic motor neurons in adult animals, we cannot rule out embryonic expression or non-cell autonomous roles in GABAergic neuronal development. Thus, to examine GABAergic motor neuron development and synapse formation, we utilized existing transgenic strains carrying either a soluble GFP or a GFP synapse marker (GFP-tagged synaptobrevin, GFP::SNB-1), each driven by a GABAergic neuron-specific promoter^32^. These transgenes were introduced into the *vav-1* mutant background. Analysis of the soluble GFP marker in *vav-1* mutants indicated that all 26 GABAergic neurons differentiated and were present in their proper locations, and circumferential axon projections (commissures) were present and did not display defects in connectivity (Fig. 4a,b). Furthermore, using the GFP::SNB-1 marker^32^, we could identify and characterize presynaptic regions between motor neurons and adjacent muscle cells in the dorsal nerve cord (DNC) (at the NMJ), which appear as fluorescent puncta (Fig. 4c). Comparing the distribution and fluorescence intensity of mutant and WT synaptic puncta can reveal defects in synaptic vesicle organization and kinetics^33^. As indicators of synaptic development and function, we used four parameters to analyse the synapses of *vav-1* mutants: the number of synaptic puncta per 20 μm of the DNC (density), the area of puncta, the fluorescence intensity of puncta and the interpunctal fluorescence intensity (intensity of the axon between synaptic puncta). From these analyses, we found *vav-1* mutant animals have a normal number of GABAergic synaptic puncta per length of the DNC (Fig. 4d, left *y* axis), and the puncta were of normal size (Fig. 4d, right *y* axis), which indicates normal development. Moreover, fluorescence intensity of puncta (Fig. 4e, left *y* axis) and fluorescence intensity of the DNC between puncta (interpunctal intensity) (Fig. 4e, right *y* axis) in *vav-1* mutants are indistinguishable from WT animals, suggesting normal synaptic activity.

To further investigate GABA motor neuron function, we utilized a pharmacologic approach. Pentylenetetrazole (PTZ), a GABA_A_ receptor antagonist, induces seizure-like behaviours in mutants with defects in GABA signalling^34^. We exposed *vav-1* mutants to PTZ and found that, unlike mutants with defects in GABA production, *vav-1* mutants did not display seizure-like behaviour (Table 1). These data indicate that GABA motor neuron activity is not impaired in *vav-1* mutants. This conclusion is further supported by the response of *vav-1* mutants to levamisole. Indeed, mutations that disrupt GABAergic motor neuron function cause levamisole hypersensitivity due to faulty inhibitory neurotransmission to the body wall muscle^30^,^31^, whereas we observed a normal response to levamisole in *vav-1* mutants (Fig. 2f). Together, these data indicate that GABAergic neurons in *vav-1* mutant animals develop normally and form proper synapses.

Since *vav-1* reporter expression was observed in the cholinergic motor neurons, we next investigated the organization of these neurons and their synapses in *vav-1* mutants. First, we analysed the development of the cholinergic nervous system using a cholinergic-specific soluble mCherry (*Punc-17::mCherry*)^35^ in the *vav-1* mutant background. From this analysis, consistent with their normal sinusoidal locomotion, we found that cholinergic motor neurons of *vav-1* mutants have normal cell positions, axon development and morphology, and were indistinguishable from WT animals (Supplementary Fig. 3). To further examine cholinergic neuron development, we investigated cholinergic synapse formation using a transgenic strain that expresses the same GFP-tagged synaptobrevin as in Fig. 4c, but specifically in cholinergic motor neurons (*Punc-129::GFP::SNB-1*)^33^. We found that, similar to GABA motor neuron synapses, synapses at cholinergic NMJs are present and appear to be organized normally in *vav-1* mutants (Fig. 4f). Examination of puncta number (Fig. 4g, left *y* axis), the size of individual synaptic puncta (Fig. 4g, right *y* axis), as well as the fluorescence intensity of puncta (Fig. 4h, left *y* axis) and interpunctal intensity (Fig. 4h, right *y* axis) in *vav-1* mutants revealed no differences from WT animals, indicating that VAV-1 does not have a role in cholinergic motor neuron synaptic development.

As *vav-1* reporters are expressed in the ALA interneuron, we also investigated the development of this neuron in *vav-1* mutants. Using a fluorescent ALA reporter, IDA-1::GFP, we found that the ALA cell body is visible in the dorsal ganglion adjacent to the nerve ring of WT and *vav-1* animals (Fig. 4i, left two panels). In addition, axonal projections that extend laterally on both sides of the animal to the posterior end are also present in WT and *vav-1* animals (Fig. 4i, right two panels). Therefore, collectively, these data indicate that VAV-1 is not required for normal neuronal development, but may instead play a role in neuronal function.

### VAV-1 is required in ALA and requires GEF function

**Figure 5:**
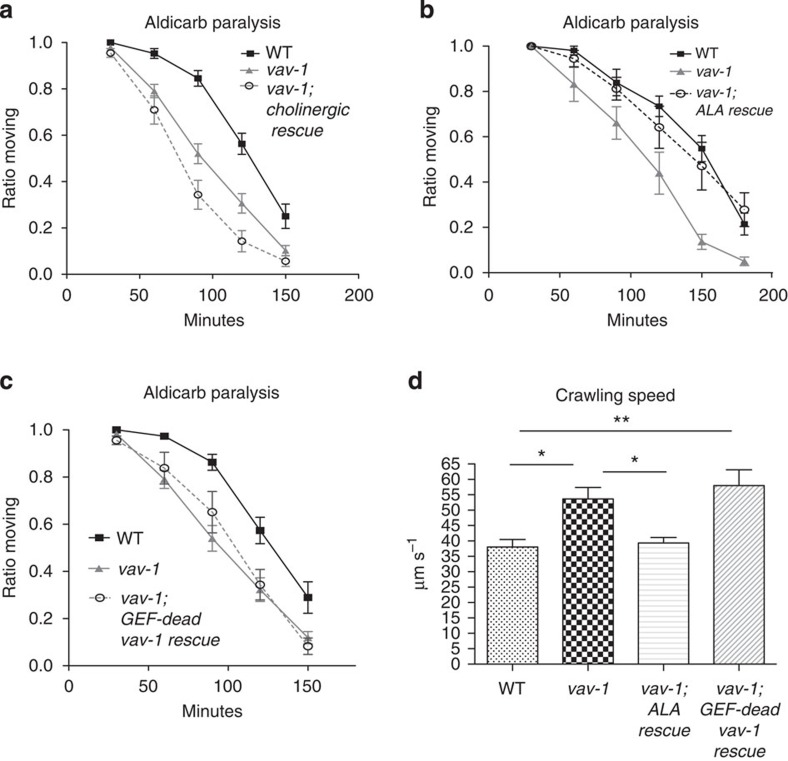
VAV-1 modulates motor neuron activity via the ALA neuron. Specific neuronal subsets were targets for VAV-1 expression in *vav-1* mutant animals to assay for rescue of aldicarb hypersensitivity (0.5 mM). (**a**) Expression of VAV-1 in cholinergic neurons does not rescue aldicarb hypersensitivity of *vav-1* mutants. (**b**) Expression in the ALA neuron rescues aldicarb hypersensitivity. (**c**) The GEF-inactive VAV-1 (*Pvav-1::vav-1 GEF-dead*) is not capable of rescuing *vav-1* mutant aldicarb hypersensitivity. In **a**–**c**, *n*=15–20 animals in ≥5 replicate assays. Data are represented as mean ±s.e.m. (statistical significance determined by two-way analysis of variance (ANOVA) and Bonferroni post-tests; more detailed information in the Methods section). (**d**) Elevated crawling speed of *vav-1* mutants is rescued by VAV-1 expression in ALA, but not by expression of GEF-dead VAV-1 under its own promoter. *n*≥20 animals in five replicate assays. Data are represented as mean ±s.e.m. (*P<0.05, **P<0.01 using one-way ANOVA and Bonferroni post-tests).

To determine which neuronal cells require VAV-1 for proper modulation of motor circuit activity, we expressed VAV-1 in different neuronal cell types using specific promoters. Since we observed strong expression with our *vav-1* reporter in ALA and variable but consistent expression in the ventral cord cholinergic motor neurons, which directly coordinate locomotion, we used the following promoters to drive *vav-1* expression in these neurons: the *ver-3* promoter to drive expression in the ALA neuron^26^ and the *unc-17* promoter to drive expression in cholinergic motor neurons^36^. To test for rescue, these neuronal transgenic strains were then crossed into the *vav-1* mutant background. For simplicity, the two genotypes are thus referred to as *vav-1; ALA rescue* and *vav-1; cholinergic rescue*. These animals were assayed for aldicarb sensitivity in comparison with WT and *vav-1* mutants. Interestingly, expression of VAV-1 in cholinergic neurons caused heightened aldicarb sensitivity in the *vav-1* mutant (Fig. 5a). Surprisingly, expression of VAV-1 in a single interneuron, ALA, returns the aldicarb sensitivity of *vav-1* mutants to a WT level (Fig. 5b). Thus, these data indicate that VAV-1 function in the ALA interneuron is sufficient for normal motor circuit activity. Since VAV-1 is a conserved GEF for the Rho GTPase family^1^,^15^, we examined whether VAV-1 requires an active GEF domain to regulate motor circuit activity. To accomplish this, a mutant *vav-1* construct that lacks GEF function^15^ was introduced into the *vav-1* mutant background. We then tested the ability of GEF-dead VAV-1 to rescue the *vav-1* mutant aldicarb hypersensitivity, and found that unlike the WT construct (Fig. 2a), the mutant GEF construct could not restore a normal aldicarb response (Fig. 5c). Therefore, these data suggest that VAV-1 activates a Rho/Rac GTPase to mediate its neuromodulatory function. Furthermore, since we observed an elevated locomotion rate in *vav-1* mutants, we measured the rate of locomotion of *vav-1; ALA rescue* animals and found that expression of VAV-1 in the ALA neuron restores normal locomotory speed to *vav-1* mutants (Fig. 5d). We also confirmed that VAV-1 lacking GEF activity cannot rescue the elevated crawling speed of *vav-1* mutants (Fig. 5d). These data indicate that VAV-1 in a GEF-dependent manner is required in ALA for normal motor circuit activity.

### ALA inhibits motor circuit activity

**Figure 6:**
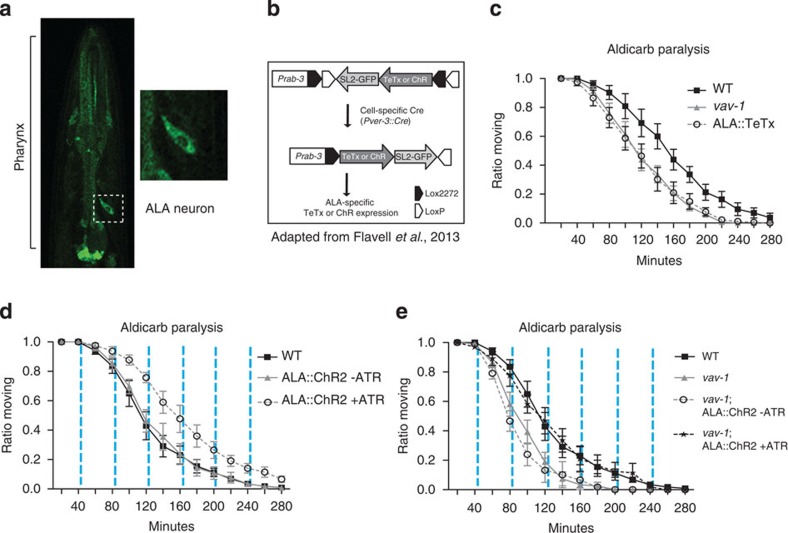
VAV-1 is required for excitation of ALA, which inhibits motor circuit activity. (**a**) Representative confocal image of functional VAV-1::GFP subcellular localization (under control of its own promoter). The left image shows VAV-1::GFP expression in the head, including the pharynx (bracket) and the ALA neuron (white dashed box). The right image shows VAV-1::GFP expression in the cell body of ALA; there was no observable fluorescence in the ALA nucleus or axons. (**b**) Schematic of the strategy used to drive ALA-specific expression of tetanus toxin (TeTx) or ChR2*(C128S), using a promoter-intersectional, inverted Cre-Lox system. (**c**) Interfering with vesicle release using ALA-specific TeTx induces aldicarb hypersensitivity, like *vav-1* mutants (0.5 mM aldicarb). (**d**) Activation of ALA using ChR2*(C128S) induces aldicarb resistance (0.5 mM aldicarb). (**e**) Activation of ALA using ChR2*(C128S), in the *vav-1* mutant background, rescues aldicarb response to wild-type sensitivity (0.5 mM aldicarb). Blue stripes indicate 1-min pulses of low-intensity blue light. *n*=15–20 animals in ≥5 replicate assays. Data are represented as mean ±s.e.m. (statistical significance determined by two-way analysis of variance and Bonferroni post-tests; more detailed information in the Methods section).

While we have found that VAV-1, via its Rho/Rac GTPase exchange activity, regulates motor circuit activity from the ALA neuron, the role(s) of VAV-1 in ALA, and of ALA in regulation of motor circuit activity, are not clear. ALA has been shown to electrically couple to the interneuron RID in the nerve ring and form chemical synapses with the command interneurons AVA, AVE (in the nerve ring) and possibly PVC (in the posterior ganglion)^37^. Thus, ALA has few synaptic partners and does not directly synapse onto motor neurons. In addition, it is not clear what neurotransmitter(s) ALA releases. However, it has been proposed that the ALA neuron is peptidergic^38^; ALA expresses proteins involved in dense-core vesicle-mediated signalling^39^. To gain insight into the function of VAV-1 in ALA, we examined the subcellular localization of a functional VAV-1::GFP expressed under its own promoter in ALA. We found that VAV-1::GFP is localized to the cell body of ALA, but not in the nucleus. Also, we did not observe VAV-1::GFP localization in the axons along the length of the animal (Fig. 6a). These data indicate that VAV-1 does not associate with synaptic or dense-core vesicles or their release sites within ALA, and suggest that VAV-1 may not have a direct role in regulating vesicle release from the ALA neuron. However, these observations do not address a potential distal role of VAV-1 in neuropeptide or neurotransmitter release from ALA.

To address whether vesicle release from ALA is involved in its modulation of motor circuit activity, we drove ALA-specific expression of tetanus toxin light chain, which blocks release of vesicles containing neurotransmitters and neuropeptides by cleaving the vesicle protein synaptobrevin^40^,^41^,^42^,^43^. To accomplish this, we exploited a cell-specific, inverted Cre-Lox expression system^44^,^45^. This approach involves one promoter driving expression of a floxed inverted tetanus toxin (TeTx) and GFP construct, and a second promoter driving Cre. Thus, by promoter overlap the expression of TeTx occurs in only one cell (Fig. 6b). We confirmed by fluorescence microscopy that GFP, and hence TeTx, is expressed solely in the ALA neuron (Supplementary Fig. 4a). Utilizing this strain, we found that blocking vesicle release by TeTx in ALA induces aldicarb hypersensitivity in otherwise WT animals (Fig. 6c) similar to *vav-1* mutants. These data suggest that ALA releases vesicles, perhaps containing neuropeptides or another neurotransmitter, to inhibit motor circuit activity.

Then, we performed a complimentary experiment, where we photoactivated the ALA neuron using ChR2. We used the same promoter-intersectional, inverted Cre-Lox strategy to drive expression of a slowly inactivating variant of ChR2*(C128S)^46^,^47^ specifically in ALA (Fig. 6b; Supplementary Fig. 4b). Owing to the length of time needed to complete an aldicarb assay, we chose this particular ChR2*(C128S) variant so that ALA activation could be prolonged beyond the duration of the blue-light stimulus, allowing us to investigate the aldicarb sensitivity of ALA-activated animals over the course of hours. Animals were exposed to a 1-min pulse of low-intensity blue light before, and every 40 min during, the aldicarb assay. Strikingly, we found that ALA-photoactivated animals are aldicarb resistant, indicating that, consistent with the ALA::TeTx results, the ALA neuron provides an inhibitory input to the motor circuit (Fig. 6d). To determine whether VAV-1 may play a role in regulating the activation state of ALA, we tested whether the aldicarb hypersensitivity of *vav*-*1* mutants could be rescued by photoactivating ALA. We found that, indeed, the *vav*-*1* mutant aldicarb response is returned to a WT sensitivity, albeit not to the level of aldicarb resistance observed in ALA::ChR2*(C128S) animals (Fig. 6e). Nevertheless, our results suggest that the role of VAV-1 in ALA is to regulate the activity of this neuron, and the effect of *vav*-*1* loss can be bypassed by photoactivating ALA.

### VAV-1 abrogates command interneurons signalling

**Figure 7:**
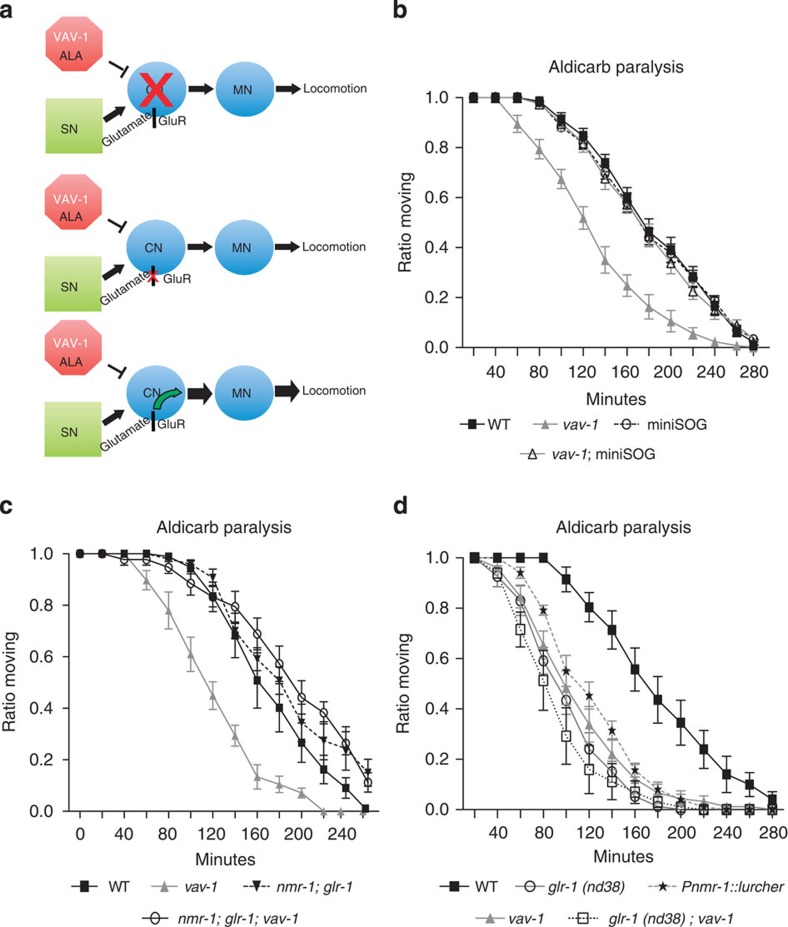
VAV-1 acts in ALA to inhibit the command interneurons of the locomotory circuit to oppose motor circuit activity. (**a**) Schematic depicting strategies to determine potential influence of command interneurons (CNs) on activity of the motor circuit, and their role in relation to VAV-1 in ALA. We used ablation of the CN with miniSOG (top panel), reduction of glutamate signalling in the CN using glutamate receptor (GluR) mutants (middle panel) and activation of glutamate signalling in the CN using gain-of-function GluR mutants (bottom panel). (**b**) Ablating the CN with miniSOG does not affect aldicarb sensitivity in a wild-type background, but prevents aldicarb sensitivity in the *vav-1* mutant background. (**c**) A double glutamate receptor mutant, *nmr-1; glr-1*, shows wild-type aldicarb response, and double *nmr-1; glr-1* mutation prevents aldicarb hypersensitivity of *vav-1* mutants. (**d**) Gain-of-function GluR mutants *glr-1 (nd38)* and *Pnmr-1::Lurcher* are aldicarb hypersensitive, like *vav-1* mutants, and double *glr-1 (nd38); vav-1* mutants are not more aldicarb hypersensitive than either mutant alone. *n*=15–20 animals in ≥5 replicate assays. Data are plotted as mean ±s.e.m. (statistical significance determined by two-way analysis of variance and Bonferroni post-tests; more detailed information in the Methods section).

To further investigate the role of VAV-1-dependent signalling from ALA in mediating motor circuit activity, we investigated the potential role of the command interneurons in regulating motor neuron activity. The command interneurons AVA, AVE and PVC are postsynaptic to ALA^37^ and promote forward and backward locomotion by directly regulating motor neurons^9^. AVA, AVE and PVC have been shown to express glutamate receptors *glr-1* and *nmr-1* (ref. 48). Since these neurons promote locomotion, we hypothesized that VAV-1-dependent signalling in ALA inhibits the activity of the AVA, AVE and PVC command interneurons, thus inhibiting motor neuron activity and locomotion. In support of this hypothesis, activated mutations in *glr-1* result in aldicarb hypersensitivity^49^. Therefore, we reasoned that if the command interneurons are a link between ALA and regulation of motor neuron activity, ablating these neurons should block the effect of *vav*-*1* loss on aldicarb sensitivity (Fig. 7a). Similarly, if we remove or reduce glutamatergic (excitatory) signalling in the command interneurons, we should decrease motor circuit activity in vav-1 mutants (Fig. 7a). Conversely, we hypothesized that if glutamate receptor signalling in the command interneurons is downstream of VAV-1 in ALA, activating glutamate receptor signalling should produce aldicarb hypersensitivity that is not synergistic with *vav-1* mutant hypersensitivity (Fig. 7a).

To test these hypotheses, we first killed the command interneurons using an existing transgenic strain carrying cell-specific miniSOG (mini singlet oxygen generator), allowing for light-inducible cell ablation^50^. We found that ablating the command interneurons (including AVA, AVD, AVE and PVC) alone does not alter aldicarb sensitivity, but returned the aldicarb response of *vav*-*1* mutants to WT sensitivity, indicating that these neurons are downstream of *vav*-*1* in the ALA neuron in regulating motor circuit activity (Fig. 7b).

Then, we generated a *glr-1; nmr-1* double loss-of-function mutant and a *glr-1; nmr-1; vav-1* triple loss-of-function mutant and tested aldicarb sensitivity. The *glr-1; nmr-1* mutant show a normal response to aldicarb (Fig. 7c). However, we found that introduction of the *glr-1* and *nmr-1* mutations into the *vav-1* mutant background completely restores a normal aldicarb response in *vav-1* animals (Fig. 7c). These data suggest that VAV-1-dependent signalling in ALA is required to abrogate excitatory glutamatergic signalling in the command interneurons and, thus, inhibit the motor circuit. Finally, we examined the aldicarb sensitivity of an activated *glr-1* mutant reported to be aldicarb hypersensitive, *glr-1(nd38)*^49^, as well as a second activated *glr-1* mutant that is equivalent to the δ2 glutamate receptor subunit mutation found in the Lurcher mouse^51^. Importantly, this second activated glutamate receptor is under control of the *nmr-1* promoter, so is expressed in fewer cells than *glr-1*, specifically including the command interneurons and two others neurons (AVG and RIM). We found that, indeed, both of these activated glutamate receptor mutants are aldicarb hypersensitive to a similar extent as *vav-1* mutants, and the *glr-1(nd38)*; *vav-1* double mutant is not significantly more aldicarb hypersensitive than either single mutant alone (Fig. 7d). In support of our hypotheses, these data indicate that glutamate receptor excitatory signalling in the command interneurons is likely downstream of the inhibitory effect of VAV-1 activity in ALA on the motor circuit.

## Discussion

Previously, VAV-1 was shown to be a critical regulator of several rhythmic activities in *C. elegans* (for example, pharyngeal pumping, ovulation and the defecation motor program)^15^,^16^,^17^. Here, we show that the rhythmic behaviour of locomotion is also abnormal in *vav-1* mutants; these animals show hyperactive locomotion due to loss of *vav-1* in the nervous system. Accordingly, *vav-1* mutants are hypersensitive to aldicarb, an ACh esterase inhibitor, which suggests heightened ACh release from the motor neurons. Furthermore, photoactivation of the cholinergic motor neurons or GABAergic motor neurons in *vav-1* mutants results in excessive muscle contraction or excessive muscle relaxation, respectively. Intriguingly, we found that expression of *vav-1* in a single interneuron ALA could rescue the locomotory and aldicarb hypersensitivity phenotypes of *vav-1* mutants. These data indicate that the ALA interneuron, in a VAV-1-dependent manner, negatively regulates motor activity. Subsequently, we found evidence that ALA is an excitable neuron that when photoactivated can repress locomotory circuit activity. In addition, photoactivation of ALA was found to bypass the loss of VAV-1 function suggesting that VAV-1 is required for ALA signalling. Moreover, we found that vesicular release from ALA is crucial for repression of motor circuit activity.

ALA is a single interneuron that is positioned slightly posterior of the nerve ring in the head. It has two processes that extend to the posterior that run along the lateral nerve cords until they reach the tail. In addition, ALA sends a short process into the DNC. Although it is believed to be peptidergic^38^, no peptides or neurotransmitters have yet been shown to have a role in ALA. In addition, ALA has few synaptic partners; ALA exhibits electrical coupling to the RID interneuron and chemical synapses with the command interneurons AVA, AVE and PVC^37^,^52^. Thus, ALA does not have a direct connection with any motor neurons. How does VAV-1 in ALA regulate motor neuron activity? Interestingly, the command interneurons are integral components of the locomotory control circuit^9^,^10^,^37^, which directly promotes the activity of the motor neurons^13^,^14^ (Fig. 1a). The motor neurons themselves are thought to be the central pattern generator that produces rhythmic sinusoidal crawling^53^ and the command interneurons promote their activity^13^,^14^. Thus, the command interneurons provide an ideal neural circuit in which ALA could participate to negatively regulate motor neuron activity and locomotion. In support of this notion, we have found that the aldicarb hypersensitivity phenotype of *vav-1* mutants can be rescued by ablation of the command interneurons or removal of excitatory glutamate receptors from the command interneurons. In addition, constitutive activation of excitatory glutamate signalling in the command interneurons leads to aldicarb hypersensitivity similar to *vav-1* mutants, and introduction of the *vav-1* mutation into the activated glutamate signalling background does not enhance the aldicarb hypersensitivity of the single mutants, suggesting they act in a common pathway. Together, the neural connectivity of ALA with the command interneurons and our genetic and pharmacological data support a model where ALA in a VAV-1-dependent manner acts to abrogate command interneuron function in promoting motor neuron activity. Previously, ALA has been found to have a role in behavioural quiescence (suppression of pharyngeal pumping and locomotion during lethargus) and it is required to suppress egg-laying activity after strong mechanical stimulation^26^,^54^. However, it is unclear how ALA suppresses these motor activities. Further studies will be required to investigate whether the motor circuit described here is involved in these other ALA-mediated behaviours.

In addition, we found that the GEF activity of VAV-1 is required for regulating motor circuit activity. Vav proteins, in addition to the Dbl homology domain, which contains the GEF activity, contain several protein–protein interaction domains, and some studies have revealed GEF-independent activity for Vav proteins, suggesting that Vav proteins can act as molecular adaptor proteins^1^,^55^,^56^,^57^. Nevertheless, our findings indicate that the role of VAV-1 in the regulation of motor circuit activity requires Rho/Rac GTPase function. The *C. elegans* Rho-family GTPases are widely expressed in most, if not all, neurons^58^,^59^ and VAV-1, *in vitro*, can promote the exchange of GDP for GTP and activate all the Rho GTPase family members in *C. elegans*^15^. Thus, one or multiple Rho-family GTPases could mediate the effects of VAV-1 in ALA.

Mammalian Vav proteins have been reported to be highly expressed in the nervous system; however, their role in neurons is not well understood. Nevertheless, analysis of Vav2 and Vav3 knockout mice has revealed a remarkable parallel with the *C. elegans vav-1* mutant (for example, nervous system hyperactivity) suggesting a conserved mechanism. Indeed, both Vav2 and Vav3 knockout mice display sympathetic nervous system hyperactivity, which results in cardiovascular and renal dysfunction^7^,^8^. However, unlike *C. elegans vav-1* mutants, which do not show any detectable nervous system developmental defects, Vav3 is required for proper GABAergic axon guidance events in the ventrolateral medulla, which is a brainstem region that regulates sympathetic nervous system activity^4^. Thus, the function of Vav3 in the mammalian nervous system has diverged or has an expanded role compared with VAV-1 in *C. elegans* in the regulation of nervous system activity. Nevertheless, the mechanism underlying the elevation in sympathetic neuronal activity in Vav2 knockout mice is not known^4^. Moreover, human Vav3 was recently identified as one of the top candidate genes in a Japanese genome-wide association study of schizophrenia^60^,^61^, indicating the importance of understanding the role of Vav proteins in the nervous system. In conclusion, our studies on VAV-1 in the *C. elegans* nervous system have demonstrated a novel role of VAV-1 in the regulation of a locomotory control circuit and indicate that *C. elegans* provides a powerful system to further explore the neuromodulatory role of Vav function in a developmentally intact nervous system.

## Methods

### *C. elegans* strains and maintenance

Animals were grown at ~20 °C on nematode growth media (NGM) plates seeded with OP50 *Escherichia coli*. Experiments were performed at ~24 °C. Strain maintenance was performed as described^62^. N2 Bristol was the WT strain. All analyses were carried out on young adult hermaphrodite worms. Strains were synchronized using hypochlorite treatment^62^ and allowed to grow (~2.5 days at 20 °C) to the young adult stage. The mutant and transgenic strains used in this study were: *glr-1 (ky176), nmr-1 (ak4), unc-25 (e156), glr-1 (nd38)*, *akEx52* [*Pnmr-1::glr-1 (A/T)*], *vav-1(ak41); akEx162* [*Ppha-4::vav-1::GFP*]*, vav-1(ak41) takIs5* [*Ppha-4::vav-1::GFP*]*, vav-1(ak41); akEx405* [*Pvav-1::vav-1::GFP, Psur-5::GFP*]*, vav-1(ak41); akIs76* [*Paex-3::vav-1::GFP*], *vav-1; akEx87* [*Pvav-1::vav-1::GFP*], *vav-1 (ak41); takEx81* [*Pvav-1::vav-1 L288Q::GFP, Pttx-3::GFP*], *takEx5* [*Pvav-1::2xNLS GFP*], *takEx6* [*Pvav-1::2xNLS mCherry*], *takEx65* [*Punc-17::vav-1::GFP, Pttx-3::GFP*], *takEx67* [*Pver-3::vav-1::GFP, Pttx-3::GFP*]*, takEx136* [*Prab-3::Inverse TeTx-SL2-GFP, Pver-3::Cre, Pttx-3::GFP*], *takEx132* [*Prab-3::Inverse ChR2 (C128S)::GFP, Pver-3::Cre, Pttx-3::GFP*]*, inIs181; inIs182* [*Pida-1::ida-1::GFP*]*, vtIs1* [*Pdat-1::GFP*], *otIs151* [*Pceh-36::RFP*]*, odIs6* [*Pglr-1::RFP*]*, juIs1* [*Punc-25::GFP::snb-1*]*, nuIs152* [*Punc-129::GFP::snb-1, Pttx-3::RFP*], *oxEx1088* [*Punc-17::halorhodopsin::GFP*], *oxIs12* [*Punc-47::GFP*], *nuIs321* [*Punc-17::RFP*]*, sid-1(pk3321) him-5(e1490) V; lin-15B(n744) X; uIs72*[*Pmyo-2::mCherry*, *Punc-119::sid-1*, *Pmec-18::mec-18::GFP*]*, zxIs3* [*Punc-47::ChR2(H134R)::YFP*], *zxIs6* [*Punc-17::ChR2(H134R)::YFP*], *juEx3771* [*Pnmr-1-tomm-20N::miniSOG; Pnmr-1-mCherry*].

### Transgenic strains and constructs

We used a standard PCR fusion-based technique^25^ to generate fluorescent *vav-1* reporters containing the full *vav-1* promoter (~8.4 kb upstream sequence), amplified from pRF35 (*vav-1* genomic clone)^15^, followed by a 2 × SV40-NLS (second-generation nuclear localization signal) and either a ~1.2-kb fragment encoding GFP [S65C], amplified from pPD135.83 (L4796), or a ~1-kb fragment encoding mCherry, amplified from pDM1284 (ref. 63), followed by the *let-858* 3′ untranslated region.

All plasmids generated to drive *vav-1* cDNA expression in specific neuronal subsets are derivatives of pRF84 [*Ppha-4::vav-1::GFP*]. The *unc-17* (~3.5 kb) promoter and the *ver-3* (~3 kb) promoter were amplified by PCR, using published primers^26^,^36^. Promoters were amplified from either genomic worm DNA or corresponding fosmids. Primers were used to flank each promoter with an SphI and an NheI restriction site, making them compatible to replace *Ppha-4* in pRF84.

To generate ALA-specific expression of tetanus toxin light chain (TeTx) and ChR2*(C128S), we used floxed/inverted vector strategy previously described^44^,^45^. Briefly, the following constructs were custom-synthesized by Genscript: *Pver-3::nCre*, *Prab-3::lox2272loxP::inverse-TeTx::SL2::GFP::inverse-lox2272loxP* and Prab-3::lox2272loxP::inverse-ChR2*(C128S)::GFP::inverse-lox2272loxP (Fig. 6b). The *ver-3* promoter (3 kb promoter^26^) drives expression in ALA, the pharyngeal saucer muscle cell and the anal sphincter muscle^26^,^64^. The *rab-3* promoter (1.2 kb promoter^36^) displays pan-neuronal expression^65^. Lox sequences^45^ and nCre sequence^43^ were obtained from published sources. The temporal and spatial overlap of these promoter elements is only in ALA.

Transgenic strains were generated by microinjection following standard protocols^66^. The injected strain for fluorescent *vav-1* reporters was *lin-15(n765ts)*. The host strain for all other transgenes was WT (N2). Listed are the injection concentrations of expression constructs of interest and coinjected DNAs: *takEx6* [*Pvav-1::2xNLS mCherry* at 100 ng μl^−1^, pJM23 at 20 ng μl^−1^, DNA ladder at 5 ng μl^−1^], *takEx65* [*Punc-17::vav-1::GFP* at 5 ng μl^−1^*, Pttx-3::GFP* at 85 ng μl^−1^, DNA ladder at 10 ng μl^−1^], *takEx67* [*Pver-3::vav-1::GFP* at 5 ng μl^−1^*, Pttx-3::GFP* at 85 ng μl^−1^, DNA ladder at 10 ng μl^−1^], *takEx75* [*Punc-25::vav-1::GFP* at 5 ng μl^−1^*, Pttx-3::GFP* at 85 ng μl^−1^, DNA ladder at 10 ng μl^−1^], *takEx81* [*Pvav-1::vav-1 L228Q::GFP* at 5 ng μl^−1^, *Pttx-3::GFP* at 80 ng μl^−1^, DNA ladder at 15 ng μl^−1^], *takEx132* [Prab-3::inverse ChR2*C128S-SL2-GFP at 25 ng μl^−1^, *Pver-3::Cre* at 25 ng μl^−1^, *Pttx-3::GFP* at 50 ng μl^−1^, DNA ladder at 25 ng μl^−1^], *takEx136* [*Prab-3::inverse TeTx-SL2-GFP* at 5 ng μl^−1^, *Pver-3::Cre* at 25 ng μl^−1^, *Pttx-3::GFP* at 62.5 ng μl^−1^, DNA ladder at 25 ng μl^−1^].

Briefly, integration of the *Ppha-4::vav-1* extrachromosomal array was carried out by treating *vav-1(ak41); akEx162 [Ppha-4::vav-1, Punc-122::GFP]*^15^ with 3,800 Rads (gamma irradiation). F2 animals were screened for 100% transmission of the *Punc-122::GFP* marker. Two integrants were isolated using this method, *takIs3* and *takIs5*, and were subsequently outcrossed 10 × using the N2 WT background.

### RNA interference

RNAi by feeding was carried out essentially as previously described^67^. In brief, L4 animals that are specifically sensitive to RNAi in the nervous system (*sid-1(pk3321) him-5(e1490) V; lin-15B(n744) X; uIs72*)^68^ were raised on bacteria expressing double-stranded RNA specific for *vav-1* and empty vector. F1 adult progeny were subjected to aldicarb assays.

### Locomotion assays

Crawling assays were conducted on NGM plates seeded with OP50. Animals were analysed as synchronized young adults on their growth plates, or young adults selected from mixed-age populations, and moved to fresh-seeded NGM plates. Each plate was placed on an Olympus dissecting microscope equipped with a Sony CCD (charge-coupled device) camera for 2–5 min before starting a video recording of locomotion. For Fig. 1b, ~5 animals were included in each 5-min recording, and 7–11 replicate plates per strain were analysed. For Fig. 5d, an estimated 20–50 animals per plate were included in each 1-min recording, and 5 replicate plates per strain were analysed. Wormlab software (MBF Bioscience) was used to calculate the average crawling speed.

### Pharmacological assays

Aldicarb and levamisole assays were performed with the experimenter blind to the strains. NGM plates containing either 1 or 0.5 mM (for differentiation between more hypersensitive strains) aldicarb were used in this study. Aldicarb assays were conducted as previously described^18^. Briefly, worms were gently prodded (with a platinum wire pick) three times between the vulva and tail, and if after any prod the worm responded by crawling, it was counted as not paralysed. Worms that crawled off the plate or exhibited vulval prolapse were removed from the assay. The ratio of worms still moving to total worms per plate was plotted for every 20- or 30-min time point. As previously observed^31^, we noted day-to-day variation in the aldicarb assays. Nevertheless, all aldicarb assay paralysis curves are the summary of at least five assays, with ~15–20 worms per genotype per plate.

Levamisole plates were made with NGM containing 100 μM drug. Worms were deemed paralysed if they failed to respond by crawling when the agar plate was dropped (from a height of a few cm) while viewing under the dissecting microscope. Plates were dropped three times, with a short pause of ~5 s in between drops. The ratio of worms paralysed to total worms per plate was plotted for every 30-min time point. Levamisole paralysis curves are the summary of at least four assays, with ~15 worms per genotype per plate.

PTZ convulsion assays were performed as previously described^34^. Worms were exposed to 6 mM PTZ on NGM plates and assayed for seizure-like behaviour at 30 and 60 min. 10-25 animals per genotype were tested, divided into three assays.

### Imaging and analysis

Animals were immobilized using 300 μM sodium azide on 2% agarose pads. Soluble fluorophore imaging to investigate neuronal structure (GABAergic GFP, cholinergic mCherry, ALA neuron GFP or functional VAV-1 GFP) was performed with a Zeiss LSM510 META-NLO confocal microscope. All other imaging was conducted with an inverted Zeiss AxioObserver microscope equipped with a Zeiss AxioCam MRm (for imaging GABA GFP::SNB-1) or Andor Clara CCD camera (for imaging cholinergic GFP::SNB-1). When comparing WT with *vav-1* mutants, 10–25 animals were analysed per genotype.

For gross morphological analysis of GABAergic neurons (*oxIs12 [Punc-47::GFP]*), cholinergic neurons (*nuIs321 [Punc-17::mCherry]*), and the ALA neuron (*inIs181; inIs182 [Pida-1::ida-1::GFP]*), z-stacks of entire worms were collected using a × 10 objective. For quantitative analysis, z-stacks of overlapping fields of view were collected, using a × 40 objective and 3-μm-thick optical slices. Image J software was used to compress *z*-stacks into maximum intensity projection images. Using these images and z-stacks, the total number of neuronal cell bodies, commissures and axonal projections were counted and analysed. The data are plotted as the mean numbers of cell bodies or commissures ±s.e.m.

For neuron identification, we investigated co-localization of known neuron reporter fluorescence with *vav-1* reporter fluorescence. Adobe Photoshop was used to overlay the fluorescent images of a *vav-1* reporter and specific neuron reporter co-expressed in one animal.

All fluorescent synaptic vesicle marker imaging was conducted with a × 63 oil objective. Images were taken of the DNC in animals with the DNC oriented towards the objective. A 20-μm-long region of interest (ROI), located 20–40 μm posterior from the vulva, was selected from each image. All intensity measurement values are in arbitrary fluorescence units, and are corrected for mean background fluorescence within the worm (background was measured for each image, near the DNC). Image J software was used to analyse GFP GABAergic synaptic vesicle distribution (*juIs1 [Punc-25::GFP::snb-1]*). Synaptic fluorescent puncta above a set intensity threshold were automatically detected using Image J, and puncta size is displayed in pixels. Metamorph software was used to analyse GFP cholinergic synaptic puncta (*nuIs152 [Punc-129::GFP::snb-1]*). Selection of ROIs and analysis of fluorescence was performed with experimenter blind to the strains. A Wacom Bamboo tablet and stylus were used to draw freehand selections around apparent puncta, interpunctal regions of the DNC and around a background ROI for each image. Metamorph software was used for fluorescence intensity and area measurements.

### Optogenetics

Optogenetic experiments were performed essentially as described^21^. All-trans retinal (ATR) plates were NGM plates seeded with 100 μl of an *E. coli* (OP50) solution containing 0.67 mM ATR and allowed to grow overnight. Control plates were seeded with 100 μl OP50 solution lacking ATR. Worms carrying cholinergic [*zxIs6 (Punc-17::ChR2)*] or GABAergic channelrhodopsin [*zxIs3 (Punc-25::ChR2)*] were grown for 96 h, or synchronized and grown from the L1 larval stage for 54 h, on ±ATR plates at 20 °C, and resulting young adult worms were picked individually to fresh, seeded NGM plates for analysis. For each worm, a 10-s video was recorded using a fluorescence dissecting microscope and Sony CCD camera. The recording was started while the worm was mid-crawl, the blue light turned on within 3 s, and left on for the duration of the video (light intensity was ~80 mW cm^−2^ from an X-Cite 120 excitation light source). From each video, one frame was selected before blue-light exposure; post light exposure, the most contracted (for *zxIs6*) or most relaxed (for *zxIs3*) frame was selected. Using Image J and a Wacom Bamboo tablet and stylus, a freehand line was traced down the centre of each worm, from nose tip to the posterior-most point of the intestine. The length of each worm before and after exposure to blue light was measured. The difference between these lengths was taken, and then divided by the starting worm length. The resulting values were either percent contraction or percent relaxation, for *zxIs6* or *zxIs3*, respectively. Worm length measurements were performed blind except for –ATR control experiments, in which worms did not visibly contract or relax.

### Optogenetic stimulation of ALA neuron for the aldicarb assay

Prolonged photoactivation of the ALA neuron was achieved by a promoter-intersectional strategy (described above) used to drive ALA-specific expression of a slowly inactivating ChR2*(C128S)^47^. Transgenic ALA::ChR2*(C128S) animals were raised from synchronized L1 larva to young adults on NGM plates seeded with OP50 bacterial solution supplemented with 0.67 mM ATR. Before beginning the aldicarb assay, each strain (including WT, *vav-1* mutant and non-ATR-treated control animals) was exposed to blue light (at ~1 mW cm^−2^) for 60 s. Immediately, animals were transferred to aldicarb plates and the experimenter assessed paralysis every 20 min, blind to the strains. After each 40-min interval, each aldicarb plate (bearing 15–20 animals) was again exposed to blue light (~1 mW cm^−2^) for 60 s to ensure prolonged activation of the channelrhodopsin. This light intensity was chosen based on previous studies using this channelrhodopsin variant (C128S) in muscle cells or neurons^45^,^47^, and the timing of blue-light exposures was chosen in an effort to maintain activation of the channelrhodopsin while minimizing a potential decrease in function due to repeated blue-light stimuli, which has been observed in muscle^47^. In addition, 60 s of blue-light stimulus per plate was about the maximum duration possible due to the time restraints imposed by the aldicarb assay (that is, at every 20 min time point,~10 min was required to test all strains for paralysis).

### Command interneuron ablation with miniSOG

Animals expressing miniSOG expressed in the command interneurons (*juEx3771* [*Pnmr-1-tomm-20N::miniSOG; Pnmr-1-mCherry*])^50^ were exposed to blue light for 30 min (~50 mW cm^−2^) as synchronized L1 larva in clear PCR tubes without caps, holding an estimated 150 animals in M9 buffer in each of four wells. Command interneuron-ablated worms were then plated onto NGM seeded with OP50 and grown to the young adult stage, along with synchronized WT and *vav-1* mutant animals, for subsequent aldicarb assays. Ablation was confirmed by loss of fluorescence in the command interneurons.

### Oil-Red-O staining

Synchronized WT and *vav-1* mutant adult animals were stained using 60% Oil-Red-O to analyse fat content, as previously described^24^. To permeabilize the cuticle, worms were resuspended and gently rocked in 120 μl of PBS and 120 μl of 2 × Modified Ruvkun Witches Brew (MRWB) buffer containing 2% paraformaldehyde for 1 h at room temperature. MRWB buffer (2 × ): 160 mM KCl, 40 mM NaCl, 14 mM Na_2_EGTA, 1 mM spermidine-HCl, 0.4 mM spermine, 30 mM Na-PIPES pH 7.4, 0.2% β-mercaptoethanol. Samples were resuspended in 60% isopropanol for 15 min to dehydrate. Animals were then stained by rocking overnight at 20 °C in 1 ml of freshly diluted and filtered (0.2 μm filter) 60% Oil-Red-O in water. Animals were washed with 200 μl 1 × PBS with 0.01% Triton X-100 to remove dye and mounted on 2% agarose pads for imaging. Whole-slide imaging at × 40 resolution was conducted using a Hamamatsu NanoZoomer and NDP.scan software (Version 2.3).

### Statistics

Any comparison made between two groups (for example, WT versus *vav-1*) was performed using a two-tailed Student’s *t*-test. For comparisons between three or more groups, we used one-way analysis of variance followed by Bonferroni post-tests. For pharmacological assay statistical analysis, we performed two-way analysis of variance followed by Bonferroni post-tests to determine differences between paralysis curves over time. The following relevant statistical comparisons were made:

In Fig. 1c, *vav-1* is significantly different than WT at 60 (*P*<0.05), 90 (*P*<0.01) and 120 min (*P*<0.05). *vav-1* is different than *vav-1; vav-1 rescue* at 90 (*P*<0.001), 120 (*P*<0.01) and 150 min (*P*<0.05). WT is not significantly different than *vav-1; vav-1 rescue*. In Fig. 1d, *vav-1* is different than WT at 60 (*P*<0.01), 90 (*P*<0.001) and 120 min (*P*<0.01). *vav-1* is different than *vav-1; neuronal rescue* at 60 (*P*<0.01) and 90 min (*P*<0.05). *vav-1; neuronal rescue* is different than WT only at 120 min (*P*<0.05). In Fig. 1e, empty vector RNAi is different than *gar-2* RNAi, positive hypersensitive control, at 90 (*P*<0.001), 120 (*P*<0.001) and 150 min (*P*<0.001). Empty vector RNAi is different than *vav-1* RNAi at 90 (*P*<0.001), 120 (*P*<0.001) and 150 min (*P*<0.001). In Fig. 1h, WT, *vav-1* and *vav-1; vav-1 rescue* are not significantly different from each other.

In Fig. 5a, WT and *vav-1* are different at 60 (*P*<0.01), 90–120 (*P*<0.001) and 150 min (*P*<0.05). WT and *vav-1; cholinergic rescue* are different at 60–120 (*P*<0.001) and 150 min (*P*<0.01). *vav-1* and *vav-1; cholinergic rescue* are different at 90 (*P*<0.01) and 120 min (*P*<0.05). In Fig. 4b,c, WT and *vav-1* are different at 120 (*P*<0.01) and 150 min (*P*<0.001). In Fig. 5b, WT is not significantly different from *vav-1; ALA neuron rescue*. Paralysis of *vav-1* versus *vav-1; ALA neuron rescue* is different at 150 (*P*<0.01) and 180 min (*P*<0.05). In Fig. 5c, WT and *vav-1* are different at 60 (*P*<0.05), 90 (*P*<0.001) and 120 min (*P*<0.01). WT and *vav-1; GEF-dead rescue* are different at 90 (*P*<0.05), 120 (*P*<0.01) and 150 min (*P*<0.05). *vav-1* and *vav-1; GEF-dead rescue* are not significantly different from each other.

In Fig. 6c, WT and *vav-1* are different at 120 (*P*<0.01), 140 (*P*<0.01), 160 (*P*<0.05) and 180 min (*P*<0.05). WT and ALA::TeTx are different at 100 (*P*<0.05), 120 (*P*<0.05), 140 (*P*<0.001) and 160 min (*P*<0.05). *vav-1* and ALA::TeTx are not statistically different. In Fig. 6d, WT and ALA::ChR2 +ATR are different at 100 (*P*<0.05), 120 (*P*<0.0001), 140 (*P*<0.001) and 160 min (*P*<0.05). ALA::ChR2 –ATR and ALA::ChR2 +ATR are different at 120 (*P*<0.001), 140 (*P*<0.05) and 160 min (*P*<0.05). WT and ALA::ChR2 –ATR are not statistically different. In Fig. 6e, WT and *vav-1* are different at 80 (*P*<0.05), 100 (*P*<0.01) and 120 min (*P*<0.05). WT and *vav-1*; ALA::ChR2 are different at 80 (*P*<0.0001), 100 (*P*<0.0001) and 120 min (*P*<0.01). *vav-1* and *vav-1*; ALA::ChR2 +ATR are different at 120 (*P*<0.01) and 140 min (*P*<0.05). The following paralysis curves were not statistically different: WT and *vav-1*; ALA::ChR2 +ATR, as well as *vav-1* and *vav-1*; ALA::ChR2 –ATR.

In Fig. 7b, WT and *vav-1* are different at 80–220 (*P*<0.001) and 240 min (*P*<0.05). The following paralysis curves are not different at any time point: WT and miniSOG, and WT and *vav-1*; miniSOG. In Fig. 7c, WT and *vav-1* are different at 80 (*P*<0.05), 100–180 (*P*<0.001) and 200 min (*P*<0.05). WT and *nmr-1; glr-1; vav-1* are different only at 220 min (*P*<0.05), and WT and *nmr-1; glr-1* are not significantly different. *nmr-1; glr-1* and *nmr-1; glr-1; vav-1* mutants are not significantly different at any point. In Fig. 7d, WT and *glr-1 (nd38)* are different at 80–200 (*P*<0.001) and 220 min (*P*<0.01). WT and *Pnmr-1::Lurcher* are different at 80 (*P*<0.05), 100–200 (*P*<0.001) and 220 min (*P*<0.01). WT and *vav-1* are different at 80–200 (*P*<0.001) and 220 min (*P*<0.05). WT and *glr-1 (nd38); vav-1* are different at 60–200 (*P*<0.001) and 220 min (*P*<0.01). *glr-1 (nd38)* and *Pnmr-1::Lurcher* are different at 80 and 120 min (*P*<0.05). *Pnmr-1::Lurcher* and *glr-1 (nd38); vav-1* are different at 60 (*P*<0.01), 80 (*P*<0.001), 100 (*P*<0.01), 120 (*P*<0.001) and 140 min (*P*<0.05). The following paralysis curves are not statistically different at any time point: *glr-1 (nd38)* and *vav-1*, *glr-1 (nd38)* and *glr-1 (nd38); vav-1*, *vav-1* and *glr-1 (nd38); vav-1*, *Pnmr-1::Lurcher* and *vav-1*.

## Additional information

**How to cite this article:** Fry, A. L. *et al.* VAV-1 acts in a single interneuron to inhibit motor circuit activity in *Caenorhabditis elegans*. *Nat. Commun.* 5:5579 doi: 10.1038/ncomms6579 (2014).

## Supplementary Material

Supplementary Figures Supplementary Figures 1-4

## Figures and Tables

**Table 1:** Response of *vav-1* mutants to 6 mM PTZ.

**Genotype**	**% Animals displaying anterior seizure (30 min)**	**% Animals displaying anterior seizure (60 min)**
WT	0	0
*unc-25 (e156)*/GAD*	70	87
*vav-1 (ak41)*	0	0

PTZ, pentylenetetrazole; WT, wild type.

^*^unc-25 encodes a glutamic acid decarboxylase.

## References

[b1] Bustelo X. R. Vav proteins, adaptors and cell signaling. Oncogene 20, 6372–6381 (2001). 11607839 10.1038/sj.onc.1204780

[b2] Turner M. & Billadeau D. D. VAV proteins as signal integrators for multi-subunit immune-recognition receptors. Nat. Rev. Immunol. 2, 476–486 (2002). 12094222 10.1038/nri840

[b3] Cowan C. W. *et al.* Vav family GEFs link activated Ephs to endocytosis and axon guidance. Neuron 46, 205–217 (2005). 15848800 10.1016/j.neuron.2005.03.019

[b4] Sauzeau V. *et al.* Vav3 is involved in GABAergic axon guidance events important for the proper function of brainstem neurons controlling cardiovascular, respiratory, and renal parameters. Mol. Biol. Cell 21, 4251–4263 (2010). 20926682 10.1091/mbc.E10-07-0639 PMC2993752

[b5] Malartre M., Ayaz D., Amador F. F. & Martin-Bermudo M. D. The guanine exchange factor vav controls axon growth and guidance during Drosophila development. J. Neurosci. 30, 2257–2267 (2010). 20147552 10.1523/JNEUROSCI.1820-09.2010 PMC6634040

[b6] Hale C. F. *et al.* Essential role for vav Guanine nucleotide exchange factors in brain-derived neurotrophic factor-induced dendritic spine growth and synapse plasticity. J. Neurosci. 31, 12426–12436 (2011). 21880903 10.1523/JNEUROSCI.0685-11.2011 PMC3183742

[b7] Sauzeau V., Jerkic M., Lopez-Novoa J. M. & Bustelo X. R. Loss of Vav2 proto-oncogene causes tachycardia and cardiovascular disease in mice. Mol. Biol. Cell 18, 943–952 (2007). 17202406 10.1091/mbc.E06-09-0877 PMC1805112

[b8] Sauzeau V. *et al.* Vav3 proto-oncogene deficiency leads to sympathetic hyperactivity and cardiovascular dysfunction. Nat. Med. 12, 841–845 (2006). 16767097 10.1038/nm1426 PMC1997289

[b9] Chalfie M. *et al.* The neural circuit for touch sensitivity in Caenorhabditis elegans. J. Neurosci. 5, 956–964 (1985). 3981252 10.1523/JNEUROSCI.05-04-00956.1985 PMC6565008

[b10] de Bono M. & Maricq A. V. Neuronal substrates of complex behaviors in C. elegans. Annu. Rev. Neurosci. 28, 451–501 (2005). 16022603 10.1146/annurev.neuro.27.070203.144259

[b11] Piggott B. J., Liu J., Feng Z., Wescott S. A. & Xu X. Z. The neural circuits and synaptic mechanisms underlying motor initiation in C. elegans. Cell 147, 922–933 (2011). 22078887 10.1016/j.cell.2011.08.053 PMC3233480

[b12] Brockie P. J. *et al.* Cornichons control ER export of AMPA receptors to regulate synaptic excitability. Neuron 80, 129–142 (2013). 24094107 10.1016/j.neuron.2013.07.028 PMC3795439

[b13] Xie L. *et al.* NLF-1 delivers a sodium leak channel to regulate neuronal excitability and modulate rhythmic locomotion. Neuron 77, 1069–1082 (2013). 23522043 10.1016/j.neuron.2013.01.018

[b14] Liu P., Chen B. & Wang Z. W. Postsynaptic current bursts instruct action potential firing at a graded synapse. Nat. Commun. 4, 1911 (2013). 23715270 10.1038/ncomms2925 PMC3683072

[b15] Norman K. R. *et al.* The Rho/Rac-family guanine nucleotide exchange factor VAV-1 regulates rhythmic behaviors in C. elegans. Cell 123, 119–132 (2005). 16213217 10.1016/j.cell.2005.08.001

[b16] Govindan J. A., Cheng H., Harris J. E. & Greenstein D. Galphao/i and Galphas signaling function in parallel with the MSP/Eph receptor to control meiotic diapause in C. elegans. Curr. Biol. 16, 1257–1268 (2006). 16824915 10.1016/j.cub.2006.05.020

[b17] Nehrke K., Denton J. & Mowrey W. Intestinal Ca2+ wave dynamics in freely moving C. elegans coordinate execution of a rhythmic motor program. Am. J. Physiol. Cell Physiol. 294, C333–C344 (2008). 17942636 10.1152/ajpcell.00303.2007

[b18] Mahoney T. R., Luo S. & Nonet M. L. Analysis of synaptic transmission in Caenorhabditis elegans using an aldicarb-sensitivity assay. Nat. Protoc. 1, 1772–1777 (2006). 17487159 10.1038/nprot.2006.281

[b19] Iwasaki K., Staunton J., Saifee O., Nonet M. & Thomas J. H. aex-3 encodes a novel regulator of presynaptic activity in C. elegans. Neuron 18, 613–622 (1997). 9136770 10.1016/s0896-6273(00)80302-5

[b20] Nagel G. *et al.* Channelrhodopsins: directly light-gated cation channels. Biochem. Soc. Trans. 33, 863–866 (2005). 16042615 10.1042/BST0330863

[b21] Liewald J. F. *et al.* Optogenetic analysis of synaptic function. Nat. Methods 5, 895–902 (2008). 18794862 10.1038/nmeth.1252

[b22] Ben Arous J., Laffont S. & Chatenay D. Molecular and sensory basis of a food related two-state behavior in C. elegans. PloS ONE 4, e7584 (2009). 19851507 10.1371/journal.pone.0007584 PMC2762077

[b23] Govorunova E. G. *et al.* A homolog of FHM2 is involved in modulation of excitatory neurotransmission by serotonin in C. elegans. PloS ONE 5, e10368 (2010). 20442779 10.1371/journal.pone.0010368 PMC2860991

[b24] O’Rourke E. J., Soukas A. A., Carr C. E. & Ruvkun G. C. elegans major fats are stored in vesicles distinct from lysosome-related organelles. Cell Metab. 10, 430–435 (2009). 19883620 10.1016/j.cmet.2009.10.002 PMC2921818

[b25] Hobert O. PCR fusion-based approach to create reporter gene constructs for expression analysis in transgenic C. elegans. Biotechniques 32, 728–730 (2002). 11962590 10.2144/02324bm01

[b26] Van Buskirk C. & Sternberg P. W. Epidermal growth factor signaling induces behavioral quiescence in Caenorhabditis elegans. Nat. Neurosci. 10, 1300–1307 (2007). 17891142 10.1038/nn1981

[b27] Nass R., Hall D. H., Miller D. M. 3rd & Blakely R. D. Neurotoxin-induced degeneration of dopamine neurons in Caenorhabditis elegans. Proc. Natl Acad. Sci. USA 99, 3264–3269 (2002). 11867711 10.1073/pnas.042497999 PMC122507

[b28] Shim J., Umemura T., Nothstein E. & Rongo C. The unfolded protein response regulates glutamate receptor export from the endoplasmic reticulum. Mol. Biol. Cell 15, 4818–4828 (2004). 15317844 10.1091/mbc.E04-02-0108 PMC524730

[b29] Johnston R. J. & Hobert O. A microRNA controlling left/right neuronal asymmetry in Caenorhabditis elegans. Nature 426, 845–849 (2003). 14685240 10.1038/nature02255

[b30] Vashlishan A. B. *et al.* An RNAi screen identifies genes that regulate GABA synapses. Neuron 58, 346–361 (2008). 18466746 10.1016/j.neuron.2008.02.019

[b31] Dabbish N. S. & Raizen D. M. GABAergic synaptic plasticity during a developmentally regulated sleep-like state in C. elegans. J. Neurosci. 31, 15932–15943 (2011). 22049436 10.1523/JNEUROSCI.0742-11.2011 PMC3226813

[b32] Jorgensen E. M. *et al.* Defective recycling of synaptic vesicles in synaptotagmin mutants of Caenorhabditis elegans. Nature 378, 196–199 (1995). 7477324 10.1038/378196a0

[b33] Sieburth D. *et al.* Systematic analysis of genes required for synapse structure and function. Nature 436, 510–517 (2005). 16049479 10.1038/nature03809

[b34] Locke C. J. *et al.* Pharmacogenetic analysis reveals a post-developmental role for Rac GTPases in Caenorhabditis elegans GABAergic neurotransmission. Genetics 183, 1357–1372 (2009). 19797046 10.1534/genetics.109.106880 PMC2784297

[b35] Chan J. P., Hu Z. & Sieburth D. Recruitment of sphingosine kinase to presynaptic terminals by a conserved muscarinic signaling pathway promotes neurotransmitter release. Genes Dev. 26, 1070–1085 (2012). 22588719 10.1101/gad.188003.112 PMC3360562

[b36] Mahoney T. R. *et al.* Intestinal signaling to GABAergic neurons regulates a rhythmic behavior in Caenorhabditis elegans. Proc. Natl Acad. Sci. USA 105, 16350–16355 (2008). 18852466 10.1073/pnas.0803617105 PMC2570992

[b37] White J. G., Southgate E., Thomson J. N. & Brenner S. The structure of the nervous system of the nematode Caenorhabditis elegans. Philos. Trans. R. Soc. Lond. 314, 1–340 (1986). 22462104 10.1098/rstb.1986.0056

[b38] Nelson M. D. & Raizen D. M. A sleep state during C. elegans development. Curr. Opin. Neurobiol. 23, 824–830 (2013). 23562486 10.1016/j.conb.2013.02.015 PMC3735717

[b39] Kim K. & Li C. Expression and regulation of an FMRFamide-related neuropeptide gene family in Caenorhabditis elegans. J. Comp. Neurol. 475, 540–550 (2004). 15236235 10.1002/cne.20189

[b40] Schiavo G. *et al.* Tetanus and botulinum-B neurotoxins block neurotransmitter release by proteolytic cleavage of synaptobrevin. Nature 359, 832–835 (1992). 1331807 10.1038/359832a0

[b41] Sweeney S. T., Broadie K., Keane J., Niemann H. & O’Kane C. J. Targeted expression of tetanus toxin light chain in Drosophila specifically eliminates synaptic transmission and causes behavioral defects. Neuron 14, 341–351 (1995). 7857643 10.1016/0896-6273(95)90290-2

[b42] Davis M. W., Morton J. J., Carroll D. & Jorgensen E. M. Gene activation using FLP recombinase in C. elegans. PLoS Genet. 4, e1000028 (2008). 18369447 10.1371/journal.pgen.1000028 PMC2265415

[b43] Macosko E. Z. *et al.* A hub-and-spoke circuit drives pheromone attraction and social behaviour in C. elegans. Nature 458, 1171–1175 (2009). 19349961 10.1038/nature07886 PMC2760495

[b44] Sohal V. S., Zhang F., Yizhar O. & Deisseroth K. Parvalbumin neurons and gamma rhythms enhance cortical circuit performance. Nature 459, 698–702 (2009). 19396159 10.1038/nature07991 PMC3969859

[b45] Flavell S. W. *et al.* Serotonin and the neuropeptide PDF initiate and extend opposing behavioral states in C. elegans. Cell 154, 1023–1035 (2013). 23972393 10.1016/j.cell.2013.08.001 PMC3942133

[b46] Berndt A., Yizhar O., Gunaydin L. A., Hegemann P. & Deisseroth K. Bi-stable neural state switches. Nat. Neurosci. 12, 229–234 (2009). 19079251 10.1038/nn.2247

[b47] Schultheis C., Liewald J. F., Bamberg E., Nagel G. & Gottschalk A. Optogenetic long-term manipulation of behavior and animal development. PloS ONE 6, e18766 (2011). 21533086 10.1371/journal.pone.0018766 PMC3080377

[b48] Brockie P. J., Madsen D. M., Zheng Y., Mellem J. & Maricq A. V. Differential expression of glutamate receptor subunits in the nervous system of Caenorhabditis elegans and their regulation by the homeodomain protein UNC-42. J. Neurosci. 21, 1510–1522 (2001). 11222641 10.1523/JNEUROSCI.21-05-01510.2001 PMC6762961

[b49] Allen A. T., Maher K. N., Wani K. A., Betts K. E. & Chase D. L. Coexpressed D1- and D2-like dopamine receptors antagonistically modulate acetylcholine release in Caenorhabditis elegans. Genetics 188, 579–590 (2011). 21515580 10.1534/genetics.111.128512 PMC3176529

[b50] Qi Y. B., Garren E. J., Shu X., Tsien R. Y. & Jin Y. Photo-inducible cell ablation in Caenorhabditis elegans using the genetically encoded singlet oxygen generating protein miniSOG. Proc. Natl Acad. Sci. USA 109, 7499–7504 (2012). 22532663 10.1073/pnas.1204096109 PMC3358873

[b51] Zheng Y., Brockie P. J., Mellem J. E., Madsen D. M. & Maricq A. V. Neuronal control of locomotion in C. elegans is modified by a dominant mutation in the GLR-1 ionotropic glutamate receptor. Neuron 24, 347–361 (1999). 10571229 10.1016/s0896-6273(00)80849-1

[b52] Altun Z. F., Herndon L. A., Crocker C., Lints R., Hall D. H. (eds) Wormatlas, http://www.wormatlas.org (2002–2012).

[b53] Wen Q. *et al.* Proprioceptive coupling within motor neurons drives C. elegans forward locomotion. Neuron 76, 750–761 (2012). 23177960 10.1016/j.neuron.2012.08.039 PMC3508473

[b54] Sanders J. *et al.* The Caenorhabditis elegans interneuron ALA is (also) a high-threshold mechanosensor. BMC Neurosci. 14, 156 (2013). 24341457 10.1186/1471-2202-14-156 PMC3878553

[b55] del Pozo M. A. *et al.* Guanine exchange-dependent and -independent effects of Vav1 on integrin-induced T cell spreading. J. Immunol. 170, 41–47 (2003). 12496381 10.4049/jimmunol.170.1.41

[b56] Hornstein I., Alcover A. & Katzav S. Vav proteins, masters of the world of cytoskeleton organization. Cell. Signal. 16, 1–11 (2004). 14607270 10.1016/s0898-6568(03)00110-4

[b57] Zeng L. *et al.* Vav3 mediates receptor protein tyrosine kinase signaling, regulates GTPase activity, modulates cell morphology, and induces cell transformation. Mol. Cell. Biol. 20, 9212–9224 (2000). 11094073 10.1128/mcb.20.24.9212-9224.2000 PMC102179

[b58] Zipkin I. D., Kindt R. M. & Kenyon C. J. Role of a new Rho family member in cell migration and axon guidance in C. elegans. Cell 90, 883–894 (1997). 9298900 10.1016/s0092-8674(00)80353-0

[b59] Struckhoff E. C. & Lundquist E. A. The actin-binding protein UNC-115 is an effector of Rac signaling during axon pathfinding in C. elegans. Development (Cambridge, England) 130, 693–704 (2003). 12506000 10.1242/dev.00300

[b60] Aleksic B. *et al.* Analysis of the VAV3 as candidate gene for schizophrenia: evidences from voxel-based morphometry and mutation screening. Schizophr. Bull. 39, 720–728 (2013). 22416266 10.1093/schbul/sbs038 PMC3627762

[b61] Ikeda M. *et al.* Genome-wide association study of schizophrenia in a Japanese population. Biol. Psychiatry 69, 472–478 (2011). 20832056 10.1016/j.biopsych.2010.07.010

[b62] Stiernagle T. Maintenance of *C. elegans*. WormBook ed. The *C. elegans* Research Community, WormBook, http://www .wormbook.org (2006).

[b63] Jensen M. *et al.* Wnt signaling regulates acetylcholine receptor translocation and synaptic plasticity in the adult nervous system. Cell 149, 173–187 (2012). 22464329 10.1016/j.cell.2011.12.038 PMC3375111

[b64] Popovici C., Isnardon D., Birnbaum D. & Roubin R. Caenorhabditis elegans receptors related to mammalian vascular endothelial growth factor receptors are expressed in neural cells. Neurosci. Lett. 329, 116–120 (2002). 12161275 10.1016/s0304-3940(02)00595-5

[b65] Nonet M. L. *et al.* Caenorhabditis elegans rab-3 mutant synapses exhibit impaired function and are partially depleted of vesicles. J. Neurosci. 17, 8061–8073 (1997). 9334382 10.1523/JNEUROSCI.17-21-08061.1997 PMC6573758

[b66] Hope I. A. C. elegans: a Practical Approach Oxford University Press (1999).

[b67] Timmons L. & Fire A. Specific interference by ingested dsRNA. Nature 395, 854 (1998). 9804418 10.1038/27579

[b68] Calixto A., Chelur D., Topalidou I., Chen X. & Chalfie M. Enhanced neuronal RNAi in C. elegans using SID-1. Nat. Methods 7, 554–559 (2010). 20512143 10.1038/nmeth.1463 PMC2894993

