# Supplementary material for: VAV-1 acts in a single interneuron to inhibit motor circuit activity in Caenorhabditis elegans
Source: Nat Commun. 2014 Nov 21;5:5579. doi: 10.1038/ncomms6579 (PMC4241504; doi:10.1038/ncomms6579)
Supplement: Supplementary Figures — 1-4 [file ncomms6579-s1.pdf]

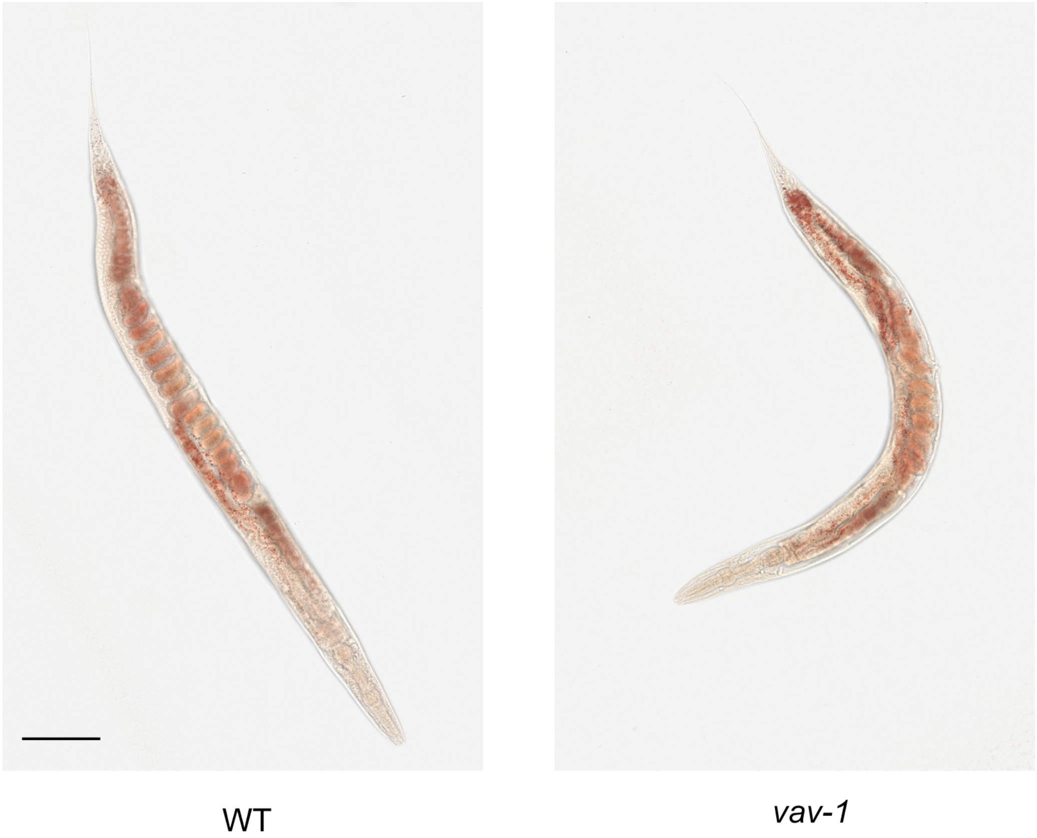

Supplementary Figure 1. Fat content of *vav-1* mutant adults is normal. Wild type (left) and *vav-1* mutant (right) adult animals were stained with Oil-Red-O to investigate fat storage. *vav-1* mutants store an approximately wild type amount of fat, indicating that these animals do not have any major defect in feeding. Anterior is pointed down. Scale bar = 100 µm.

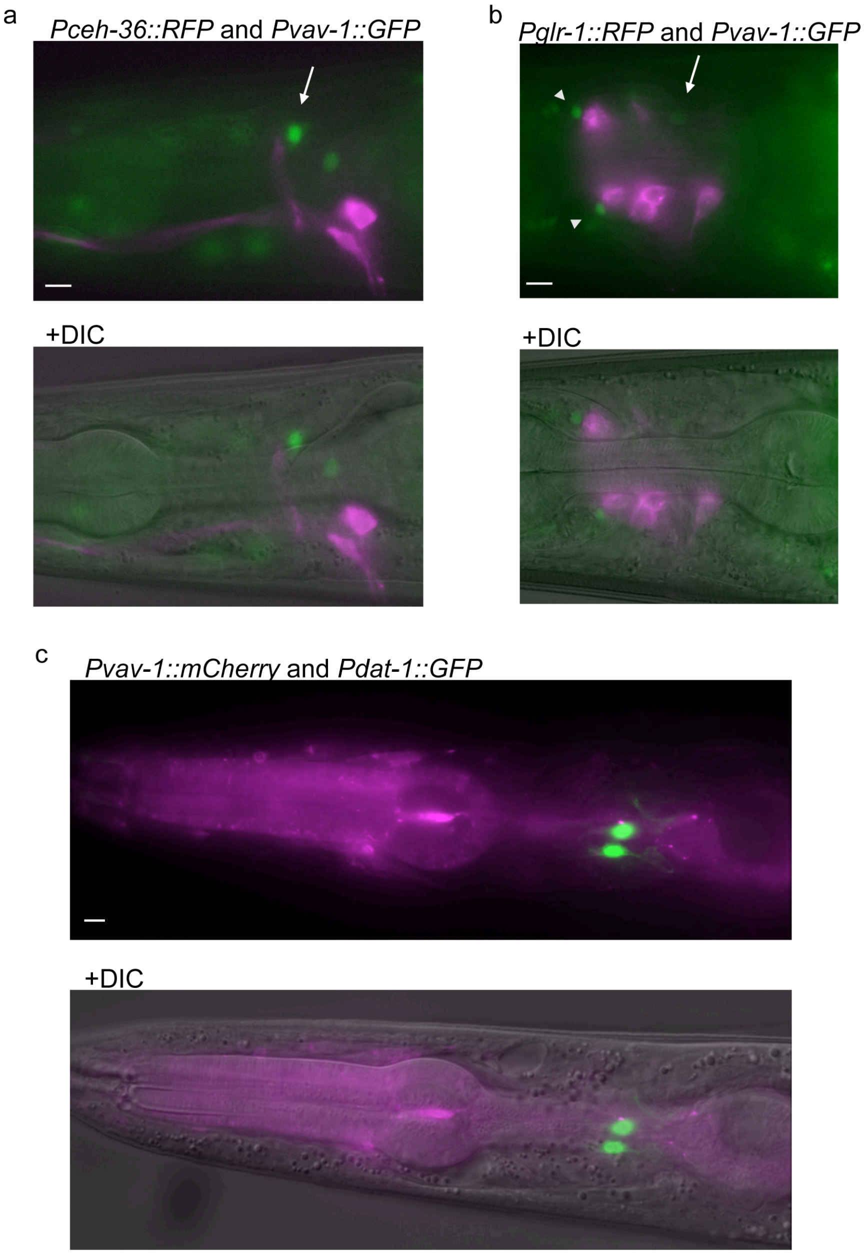

Supplementary Figure 2. *vav-1* reporters are not expressed in a subset of sensory neurons, command interneurons, or dopaminergic neurons. (**a**) A *vav-1::GFP* (shown in green) reporter does not colocalize with a marker of AWC and ASE sensory neurons (*Pceh-36::RFP*, shown in purple). (**b**) A *vav-1::GFP* reporter does not colocalize with a marker of command interneurons (*Pglr-1::RFP*, shown in purple). Arrows in (**a**) and (**b**) mark the ALA neuron, and arrowheads mark RMED and RMEV. (**c**) A *vav-1::mCherry* reporter (shown in purple) does not colocalize with a marker of dopaminergic neurons (*Pdat-1::GFP*, shown in green). Anterior is to the left. Scale bars = 5 μm.

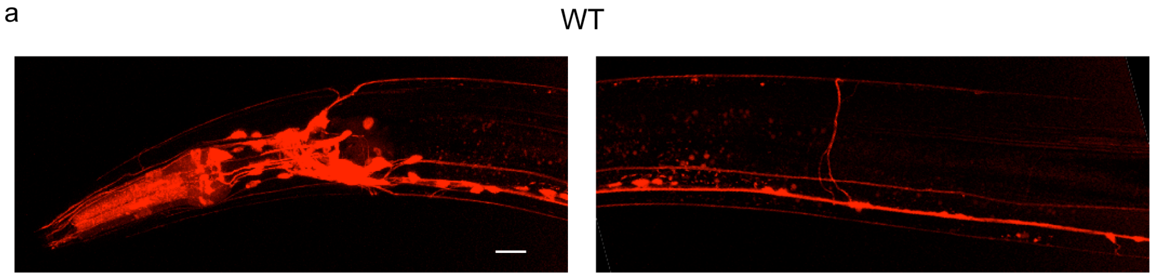

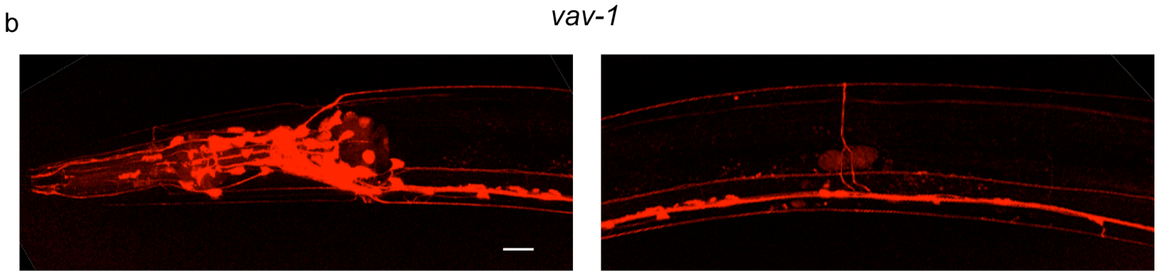

Supplementary Figure 3. Morphology of cholinergic neurons in *vav-1* mutants is normal. A cholinergic neuron reporter (*Punc-17::RFP*) was used to investigate the overall structure of these neurons in (**a**) WT and (**b**) *vav-1* mutant adult animals. The images on the left show the head of single animals, and images on the right show the corresponding midsection. No major differences were observed in cell body or axon morphology. Anterior is to the left. n=3 (WT) and 5 (*vav-1*). Scale bars = 10 μm.

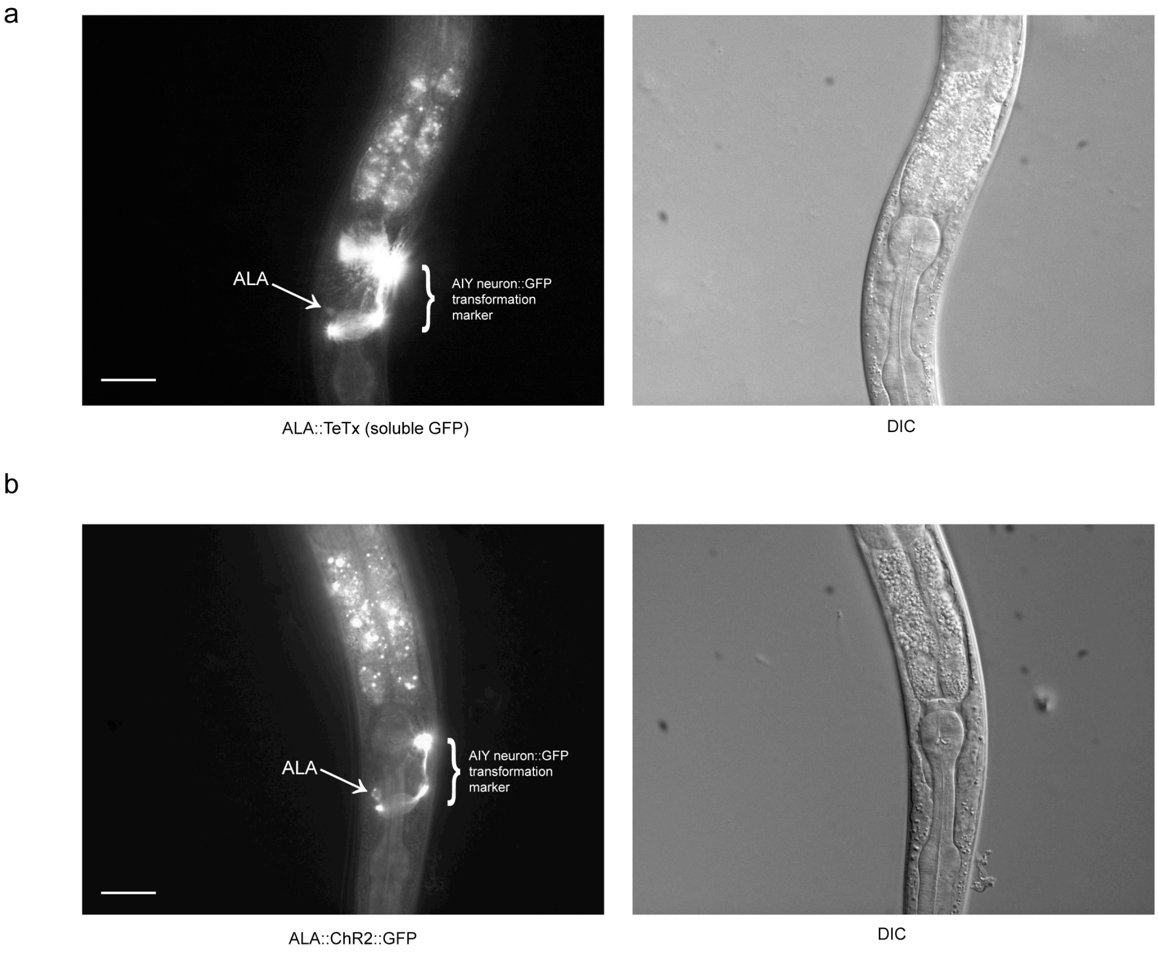

Supplementary Figure 4. Tetanus toxin (TeTx) and ChR2*(C128S) are expressed specifically in the ALA neuron.(**a**) Fluorescence microscopy of transgenic animals expressing ALA neuron-specific TeTx and soluble GFP, or (**b**) ALA neuron-specific ChR2*(C128S)::GFP (left panels). Arrows indicate ALA neuron GFP, identified by cell position just posterior to the nerve ring, and cell shape. GFP indicates expression of the TeTx or ChR2*(C128S) construct. Brackets highlight the much brighter transformation marker, AIY neuron::GFP (*Pttx-3::GFP*). Also present in the images is intestinal autofluorescence (gut granules). Other neurons were not observed to express GFP. Paired with the fluorescent images are DIC images of the same focal plane (right panels). Anterior is pointed down. Scale bars = 20 μm.
